# Discovery of the antifungal compound ilicicolin K through genetic activation of the ilicicolin biosynthetic pathway in *Trichoderma reesei*

**DOI:** 10.1186/s13068-025-02628-3

**Published:** 2025-03-11

**Authors:** Isabella Burger, Matthias Schmal, Kathrin Peikert, Lukas Fourtis, Christoph Suster, Christian Stanetty, Dominik Schnalzer, Barbara Hufnagel, Thomas Böttcher, Ruth Birner-Gruenberger, Robert L. Mach, Astrid R. Mach-Aigner, Matthias Schittmayer, Christian Zimmermann

**Affiliations:** 1 Institute of Chemical Technologies and Analytics, TU Wien, 1060 Vienna, Austria; 2 Institute of Chemical, Environmental and Bioscience Engineering, TU Wien, 1060 Vienna, Austria; 3 Institute of Applied Synthetic Chemistry, TU Wien, 1060 Vienna, Austria; 4 Institute for Biological Chemistry & Centre for Microbiology and Environmental Systems Science, University of Vienna, 1090 Vienna, Austria

**Keywords:** Natural products, Multi-omics, Fungi, Antifungal agents, Pathway engineering

## Abstract

**Background:**

Given the global rise in antimicrobial resistance, the discovery of novel antimicrobial agents and production processes thereof are of utmost importance. To this end we have activated the gene cluster encoding for the biosynthesis of the potent antifungal compound ilicicolin H in the fungus *Trichoderma reesei*. While the biosynthetic gene cluster (BGC) is silent under standard cultivation conditions, we achieved BGC activation by genetically overexpressing the transcription factor TriliR.

**Results:**

Successful activation was confirmed by RT-qPCR, proteomic and metabolomic analyses. Metabolomic profiling upon BGC expression revealed high-yield production of ilicicolin H. To elucidate the enzymatically highly diverse functionality of this BGC, we employed a combination of overexpression and deletions of individual genes in the BGC. While we hardly observed any of the previously reported side- or shunt products associated with heterologous ilicicolin H expression, we discovered that *Trichoderma reesei* produces a novel member of the ilicicolin family using a metabolomic molecular networking approach. This new compound, ilicicolin K, is expressed in substantial amounts in the genetically engineered *Trichoderma reesei*. Ilicicolin K differs from ilicicolin H in its structure by a second hydroxylation of the tyrosine derived phenol and an additional ring formed by an intramolecular ether bridge of the hydroxyl group at the pyridone towards the tyrosine moiety of the molecule. Bioactivity tests of ilicicolin K revealed a strong antifungal activity against *Saccharomyces cerevisiae* and a moderate activity against the human pathogen *Candida auris*, an emerging multi-drug resistant fungus.

**Conclusions:**

By activating a silent BGC in *T. reesei*, we obtained a high-yielding strain for the production of the antifungal compounds ilicicolin H and the novel ilicicolin K. These two compounds share some structural properties and are thus highly likely to act on the fungal cytochrome bc1 complex, a component of the mitochondrial repository chain. However, they possess different bioactive properties, which might suggest that ilicicolin K may overcome certain limitations of ilicicolin H.

**Graphical Abstract:**

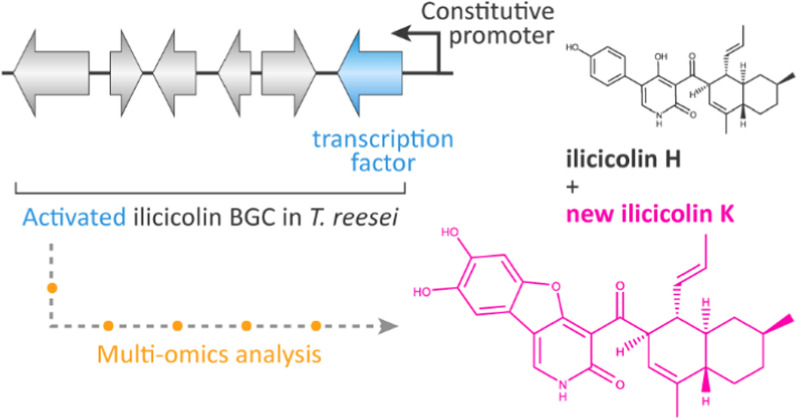

**Supplementary Information:**

The online version contains supplementary material available at 10.1186/s13068-025-02628-3.

## Background

**Fig. 1:**
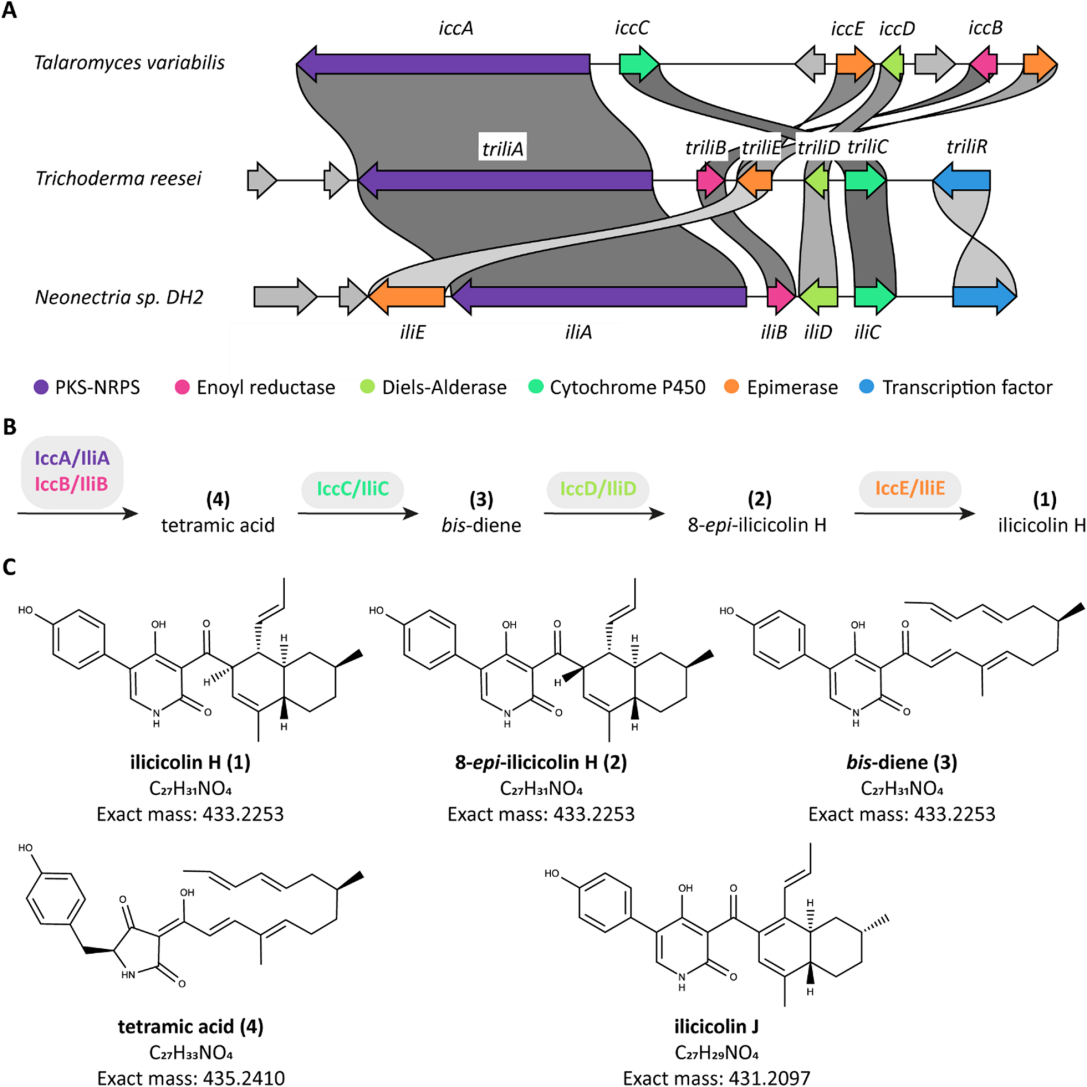
**A** The ilicicolin BGCs of *Trichoderma reesei* QM6a, *Talaromyces variabilis* HXQ-H-1, and *Neonectria* sp. DH2 were compared using the clinker tool [15]. The input.gbk files are provided in the supplemental materials. **B** Model for the biosynthetic pathway for ilicicolin H, described by Zhang *et* *al.* [12] and partially consistent with Lin *et* *al.* [13] and Shenouda *et* *al.* [14]. **C** Previously described and for this publication relevant ilicicolin compounds; **(**1**)**–**(**4**)** from Zhang *et al.* [12], ilicicolin J from Lin *et* *al.* [13]

Since ancient times, mankind has valued natural products for their therapeutic potential. While plants have long been recognized for their medicinal value, fungi, although often overlooked, harbor a remarkably diverse reservoir of potentially beneficial compounds. Among them is the natural product ilicicolin H (Fig. 1, **(1)**), which was discovered in 1971 as an antibiotic from the ascomycete *Cylindrocladium ilicicola* [1].

Known as a potent and broad-spectrum antifungal compound [2] that specifically inhibits the cytochrome bc1 complex (reductase) [3], the agent was also applied recently in several in vitro studies for the treatment of various cancer cell lines (DLD-1 colon adenocarcinoma and A549 non-small cell lung carcinoma [4], PC-3 and 22Rv1 prostate carcinoma [5], Huh7 and HepG2 hepatocellular carcinoma [6]). However, and notably, ilicicolin H exhibits a manifold higher potency against fungi, with half maximal inhibitory concentrations (IC_50_) of 2–3 ng mL^−1^ for *C. albicans* MY1055 NADH:cytochrome bc1 reductase compared to 2000 – 5000 ng mL^−1^ for rat liver cytochrome bc1 reductase [7]. Although ilicicolin H appeared as a promising treatment for fungal infections, it only showed modest efficacy in in vivo mouse models, which was attributed to high plasma protein binding [2]. Several attempts were undertaken to improve the efficacy by chemically modifying its structure, however, with limited success [2, 7, 8].

Ilicicolin H is produced by several fungi during cultivation, e.g., *Cylindrocladium ilicicola* (strain MFC-870) [1, 9, 10], *Gliocladium roseum* [2, 11], *Nectria* sp. B13 [12], and *Neonectria* sp. DH2 [13]; and many additional fungi seem to possess the corresponding genes [12]. The biosynthetic gene cluster (BGC) of ilicicolin H was discovered by two groups independently in 2019. Zhang *et al.* [12] and Lin *et al.* [13] heterologously expressed the BGCs from *Talaromyces variabile* and *Neonectria* sp. DH2, respectively, in *Aspergillus nidulans*. Recently, Shenouda *et al.* [14] heterologously expressed the ilicicolin H BGC from *Trichoderma reesei* in *A. oryzae*. The BGCs of all three fungi contain similar genes with a polyketide synthase-nonribosomal peptide synthetase (PKS-NRPS) core gene (Fig. 1A and Table 1). Please note the different sizes of the epimerase genes are likely due to a mistake of the gene model. The prediction for *Neonectria iliE* contains a TIM-like epimerase domain as well as a DnaJ-class molecular chaperone domain.

**Table 1:** Proteins of the ilicicolin H BGC from *T.* *reesei* in comparison to the homologs in *Neonectria* sp. DH2 [13] and *T. variabilis* [12]

Protein designation	Enzyme function	JGI protein ID in *T. reesei* QM6a/RutC30	Homolog in *Neonectria* sp. DH2 (% identity)	Homolog in *T. variabilis* HXQ-H-1 (% identity)
TriliA	PKS-NRPS	58285/128011	IliA (63%)	IccA (59.8%)
TriliB	Enoyl reductase	58289/74247	IliB (65.3%)	IccB (68.6%)
TriliC	Cytochrome P450	58953/33667	IliC (70.7%)	IccC (69.4%)
TriliD	Diels-Alderase	–/75073	IliD (55.6%)	IccD (51.8%)
TriliE	Epimerase	76204/96728	IliE (52%)	IccE (55.9%)
TriliR	Transcription factor	72993/74475	Pred. TF (48.5%)	n/a

Independent of the source organism, the composition of the ilicicolin H BGC is in wide parts conserved, sharing a common model (Fig. 1B) for the biosynthetic pathway: first, the polyketide synthase (PKS) part of IccA/IliA assembles the polyketide backbone, supported by the enoyl reductase IccB/IliB, and adds the methyl groups. The nonribosomal peptide synthetase (NRPS) part of IccA/IliA then adds a tyrosine. Following a Dieckmann condensation, a tetramic acid intermediate **(4)** is released. This moiety is converted to the pyridone part of ilicicolin H by the cytochrome P450 IccC/IliC via a ring expansion. Next, the Diels-Alderase IccD/IliD was suggested to catalyze the intramolecular Diels–Alder reaction yielding the decalin moiety of ilicicolin H. Up to this step, the results of the three groups concur.

Lin *et* *al.* [13] and Shenouda *et* *al.* [14] reported that these four enzymes from *Neonectria* sp. DH2 and *T. reesei*, respectively, are sufficient to obtain ilicicolin H in their heterologous expression experiments, whereas Zhang *et* *al.* [12] demonstrated that IccE is necessary to catalyze the epimerization of 8-*epi*-ilicicolin H **(2)** to ilicicolin H **(1)** in vivo and in vitro. Lin *et* *al.* [13] speculated that IccD and IliD differ, resulting in the formation of 8-*epi*-ilicicolin H and ilicicolin H, respectively. The epimerization appears to be pH-dependent [12] and the observed epimerization differences might thus alternatively be a result of varying cultivation and/or extraction conditions. Further, the possibility that a host enzyme might also carry out the epimerization has to be considered. It is unclear whether Zhang *et* *al.* [12] and Lin *et* *al.* [13] used the same *A. nidulans* strain, since Lin *et* *al.* [13] did not specify a strain designation. Importantly, there were other differences observed in the studies of these two groups that might be the result of different hosts. Most prominently, the heterologous expression conducted by Lin *et* *al.* [13] did not only produce ilicicolin H, but also a shunt product with comparable antifungal activities, ilicicolin J (Fig. 1C). Similarly, Shenouda *et* *al.* [14] reported on several acetylated side products during the heterologous expression of the *T. reesei* BGC in *A. oryzae* which they explained as a result of the native metabolism of the production host.

Herein, we describe the high-yield expression of ilicicolin H by genetic BGC activation in a native host. To this end, we overexpressed the BGC transcription factor, TriliR, under the constitutive *tef1* promotor [16] in *T. reesei*. Further, we deleted the genes *triliA* and *triliE* in the activated strain. Using a multi-omics approach including transcript analyses, proteomics, and metabolomics, we aimed at shedding further light on the biosynthesis process in the native host *T. reesei*, thus allowing a comparison to previous heterologous expression studies. Moreover, by applying metabolic molecular networking, we uncovered a novel antifungal compound, which we termed ilicicolin K.

## Material and methods

### Material

Unless otherwise specified, all chemicals were purchased from Sigma-Aldrich (St. Louis, MO, USA). Phenol was purchased from AppliChem (Darmstadt, Germany). Malt extract, peptone, isoamylalcohol, FeSO_4_, CoCl_2_·6H_2_O, Na_2_MoO_4_·H_2_O, (NH_4_)_2_SO_4_ and KH_2_PO_4_ were acquired from Merck (Darmstadt, Germany). Isopropanol, MgSO_4_·7H_2_O, CaCl_2_·2H_2_O, Na_2_HPO_4_·2H_2_O and uridine were obtained from Carl Roth (Karlsruhe, Germany). Agar and chloroform were purchased from VWR (Radnor, PA, USA). LC–MS grade acetonitrile (ACN) was acquired from VWR chemicals (Radnor, PA, USA). LC–MS grade methanol (MeOH) was obtained from Honeywell (Muskegon, MI, USA). LC–MS grade 2-propanol (IPA) was obtained from Fisher Scientific (Hampton, NH, USA). HPLC grade diethyl ether was obtained from Sigma-Aldrich (St. Louis, MO, USA). Water (H_2_O) was purified in-house using a Barnstead™ Smart2Pure™ Water Purification System from Thermo Fisher Scientific (Waltham, MA, USA). Ilicicolin H (CAS #12,689–26-8) standard, Catalog #10–3243 (Lot #X107453), was purchased from Focus Biomolecules (Plymouth Meeting, PA, USA).

Mycelium samples were lysed using the Bead Mill Max Homogenizer in combination with Tough Microorganism Lysing Mix Glass Beads (both VWR International, Radnor, PA, USA). Sonication was carried out using a Branson SFX550 sonifier from Emerson (Ferguson, MO, USA). Isolation of ilicicolin H from the medium was conducted using the Supelclean™ LC-18 SPE Tubes (Merck, Darmstadt, Germany).

Four biological replicates of fungal mycelium or culture supernatant were processed for each of the constructed and utilized strains, resulting in a total of four samples per experimental condition, unless otherwise noted.

### Strains and cultivation conditions

All *T. reesei* strains (Table 2) were maintained on malt extract (MEX) plates (3% malt extract, 0.1% peptone, 1.5% agar). 5 mM uridine or 100 mg L^−1^ Hygromycin B (Millipore, 400,051) were added if required. For liquid cultivations, 10 [9] spores L^−1^ were inoculated in Mandels Andreotti (MA) medium [17] (KH_2_PO_4_ 2 g L^−1^, (NH_4_)_2_SO_4_ 1.4 g L^−1^, urea 0.3 g L^−1^, FeSO_4_·7H_2_O 0.005 g L^−1^, MnSO_4_·H_2_O 0.0016 g L^−1^, ZnSO_4_·7H_2_O 0.0014 g L^−1^, CoCl_2_ 0.002 g L^−1^, MgSO_4_·7H_2_O 0.3 g L^−1^, CaCl_2_ 0.3 g L^−1^, peptone 0.75 g L^−1^, carbon source 10 g L^−1^) and incubated at 30 °C at 180 rpm.

**Table 2:** Utilized strains

Strain designation	Genotype	Source
QM6a Δmus53, “wildtype”	Δ*mus53*	Steiger *et* *al.* [18]
QM6a Δpyr4	Δ*mus53* Δ*pyr4*	Derntl *et* *al.* [19]
QM6a OETriliR	Δ*mus53 Ptef*::*triliR*::*Tcbh2* (upstream of *pyr4)*	This study
QM6a ΔTriliA	Δ*triliA*::*hygR* in QM6a OETriliR	This study
QM6a ΔTriliE	Δ*triliE*::*hygR* in QM6a OETriliR	This study

### Genetic constructions

All PCRs were performed with the Q5 High-Fidelity DNA Polymerase (NEB, Ipswich, MA, USA) according to the manufacturer’s instructions. For cloning purposes, the *Escherichia coli* strain Top10 (Invitrogen) and the *Saccharomyces cerevisiae* strain WW-YH10 (ATCC 208405) were used. All plasmids and genetic constructs were verified by sequencing at Microsynth (Balgach, Switzerland).

For the overexpression of TriliR, the plasmid pRP4-OETriliR was constructed. First, a NotI-free coding region of *triliR* was constructed by a SOE-PCR (Splice by Overlap Extension PCR) using the primers TriliR_fwd-AflII, TriliR_MRev_SOE, TriliR_MFwd_SOE, and TriliR_rev-SpeI and chromosomal DNA of *T. reesei* QM6a Δmus53 as template yielding a coding region with a silent mutation at R227. This modified coding region was inserted into pRP4-TX(WT) [20] via digestion with AflII and SpeI (both NEB) and ligation with T4 DNA Ligase (NEB). Approx. 20 µg of the plasmid were linearized with NotI, precipitated with sodium acetate and ethanol and dissolved in 15 µL ddH_2_O before the transformation into *T. reesei* (Figure S1).

For the construction of the *triliA* deletion cassette, yeast recombinational cloning was performed using the lithium acetate method [21] for yeast transformation. The plasmid pRS426 [22] was linearized by digestion with KpnI and HindIII. The flanking regions were amplified by PCR with the primers 5_TriliA_fwd-pRS426 and 5_TriliA_fwd-pRS426 or 3_TriliA_fwd-hph and 3_TriliA_rev-pRS426 using chromosomal DNA of *T. reesei* QM6a Δmus53 as template. The *hph* gene was amplified by PCR with the primers hph_fwd-5_TriliA and hph_rev-3_TriliA using the plasmid pAN7-1 [23] as template. The plasmid was extracted from yeast using the Zymoprep Yeast Plasmid Miniprep Kit (Zymo Research) and transformed into *E. coli* Top10 for amplification. To delete *triliA*, a split marker approach was used (Figure S2). To this end, a fusion of the 5’flank and a part of the *hph* gene were amplified by PCR using the primers 5_TriliA_fwd-pRS426 and hph_MR and the plasmid as template. Accordingly, the remaining part of the *hph* gene was amplified together with the 3’flank using the primers hph_MF and 3_TriliA_rev-pRS426. Several PCR reactions were pooled, precipitated with ethanol and dissolved in 15 µL ddH_2_O.

For the deletion of *triliE*, also a split marker approach (Figure S3) was used, but the fusion products were directly constructed by SOE-PCRs. For the *hph* gene (from pAN7-1 [23]) fragments, the primers PgpdA_fwd, hph_MR, hph_MF, and TtrpC_rev were used. The flanking regions were amplified by PCR with the primers TriliE_5fwd, TriliE_5rev-hph, TriliE_3fwd-hph, and TriliE_3rev using chromosomal DNA of *T. reesei* QM6a Δmus53 as template. The fusion PCR products were cloned into pJET1.2 using the CloneJET PCR Cloning Kit (Thermo Scientific). Before transformation the fusion products were amplified by PCR, several PCR reactions were pooled, precipitated with ethanol and dissolved in 15 µL ddH_2_O.

### Fungal transformation

*T. reesei* was transformed using a polyethylene glycol-mediated transformation protocol of protoplasts. Spores of the recipient strain were plated on sterile cellophane sheets laid on MEX plates at 30 °C overnight. The mycelium was scraped off and transferred into 15 mL Buffer A (1.2 M sorbitol, 100 mM KH_2_PO_4_, pH 5.6) containing 600 mg Vinotaste Pro (Novozymes, Bagsværd, Denmark) and 0.5 mg chitinase from *Streptomyces griseus* (Sigma-Aldrich C6137). This mixture was incubated in a sterile petri dish in an orbital shaker at 60 rpm and 30 °C for approx. 2–3 h until the mycelium was completely disintegrated. The suspension was filtered through a 70-µm cell sieve and incubated on ice for 5 min. The suspension was filled up to 40 mL with ice-cold 1.2 M sorbitol and centrifuged at 2,500 g at 4 °C for 10 min. The pellet was resuspended in 30 mL 1.2 M sorbitol and again centrifuged. The protoplasts were finally resuspended in 1 mL ice-cold Buffer B (1 M sorbitol, 25 mM CaCl_2_, 10 mM Tris–HCl, pH 7.5). Next, the DNA (either 20 µg linearized plasmid or 5 µg of fusion PCR products each for the split marker approach) was filled up to 150 µL with ice-cold Buffer B, carefully mixed with 100 µL of the protoplast suspension and 100 µL “20% PEG” (mixture of 6.7 mL Buffer B and 3.3 mL “60% PEG” (60 g PEG 4000, 1 mL 1 M Tris–HCl pH 7.5, 1 mL 1 M CaCl_2_, 38 mL ddH_2_O)) in a 50-mL reaction tube. This mixture was incubated on ice for 30 min before “60% PEG” was added in steps (50 µL, 200 µL, 500 µL). Next, the tube was incubated at room temperature for 20 min, and finally, Buffer C (1 M sorbitol, 10 mM Tris–HCl, pH 7.5) was added in steps (200 µL, 400 µL, 1 mL, 2.5 mL). For plating, the tube was filled up to 50 mL with molten, 50 °C-warm selection medium (containing 1 M sucrose) and poured into a 14.5 cm petri dish. For insertion of the TriliR overexpression construct together with *pyr4* a minimal medium was used (MA medium with glucose without peptone, pH 5.8). For the deletion of *triliA* and *triliE* by replacement with *hph*, MEX medium with hygromycin was used. The plates were incubated at 30 °C under light until colonies were visible (up to a week). The candidates were then homokaryon selected by spore streaking on selection plates.

### DNA extraction and genotyping

Mycelium was harvested and pressed dry between two sheets of filter paper. Approximately 50 mg were lysed in 1 mL CTAB buffer (1.4 M NaCl, 100 mM Tris–HCl pH 8.0, 10 mM EDTA, 2% CTAB, 1% polyvinylpyrrolidone) with 0.37 g small glass beads, 0.25 g medium glass beads and one large glass bead in a 2 mL screw cap reaction tube using a Fast-Prep-24 (MP Biomedicals, Santa Ana, CA, USA) at 6 m s^−1^ for 30 s. The samples were incubated at 65 °C for 20 min and finally centrifuged at 12,000*g* for 10 min. The supernatant was transferred to a 2-mL reaction tube and the DNA was purified by a phenol–chloroform–isoamyl alcohol extraction, followed by two chloroform extractions. The samples were then treated with RNase A (Thermo Fisher Scientific) according to the manufacturer’s instructions and the DNA finally precipitated using isopropanol and dissolved in 10 mM Tris–HCl pH 8.0.

All PCR reactions for genotyping were performed with OneTaq DNA Polymerase (NEB) according to the manufacturer’s instructions.

### RNA extraction and RT-qPCR analyses

Mycelium was harvested and pressed dry between two sheets of filter paper, frozen in liquid nitrogen, and stored at − 80 °C for up to a week. Approx. 50 mg of mycelium were disrupted in 1 mL RNAzol RT with 0.37 g small glass beads, 0.25 g medium glass beads and one large glass bead in a 2-mL screw cap reaction tube using a Fast-Prep-24 (MP Biomedicals) at 6 m s^−1^ for 30 s. The samples were centrifuged at 12,000 g for 10 min, the supernatant was transferred to a 1.5-mL reaction tube and mixed with ethanol 1:1. The RNA was purified using the Direct-zol RNA MiniPrep Kit (Zymo Research, Irvine, CA, USA) according to the manufacturer’s instructions. Notably, this kit contains a DNase treatment step. The total RNA was reverse transcribed using the LunaScript RT SuperMix Kit (NEB) according to the manufacturer’s instructions. The cDNA was diluted 1:50 in ddH_2_O and 2 µL were used as template in a 15-µL reaction using the Luna Universal qPCR Master Mix (NEB) on a Rotor-Gene Q (Qiagen, Venlo, Netherlands). Primers were added and PCR reaction conditions were chosen according to the manufacturer’s instructions. To calculate the relative transcript abundance, we used the Pfaffl method [24] and the *act1* and *sar1* genes as reference genes [25].

### Sample preparation for HPLC(–MS/MS) and NMR analysis

#### Ilicicolin H standard

100 µg of ilicicolin H standard were dissolved in 100 µL of DMSO for a final concentration of 1 µg µL^−1^. 1 µL of this stock was diluted with 99 µL of 50% ACN, to obtain a final concentration of 0.01 µg µL^−1^. For LC–MS analysis, 1 µL was injected.

#### Polar extracts of mycelium

100 mg of frozen mycelium sample were weighed into glass bead-milling tubes and 1 mL of polar extract composed of a mixture of acetonitrile/methanol/water (40:40:20) were added. Lysis was conducted by bead-milling (4 × 30 s, 6 m s^−1^) and subsequent sonication (30 s, 10% intensity). The lysed samples were centrifuged for 10 min at 20,000 *g* and 20 °C and the supernatant was transferred into fresh Eppendorf tubes. The supernatants were centrifuged again, for 1 min at 20,000 *g*. For analysis, 10 µL of the supernatant were taken, diluted in 90 µL of 50% ACN and 1 µL was injected for LC–MS analysis.

#### Quantification of ilicicolin H in medium

For quantification of ilicicolin H in the media, a matrix-matched external calibration curve was utilized. For that, 4 mL of culture medium of each wildtype and ΔTriliA quadruplicate (*n* = 8 in total) were pooled, which do not contain ilicicolin H in detectable quantities (below LOD of 21.4 ng mL^−1^, Figure S5). 6 × 2 mL were taken out and each spiked with precise amounts of ilicicolin H standard (50 ng, 100 ng, 200 ng, 500 ng, 1000 ng and 2000 ng). These standards and the culture supernatants of the other 8 samples (4 × OETriliR, 4 × ΔTriliE, 50 mL each) were cleaned up using C18-SPE columns. The elution buffer was constituted of 10% acetonitrile in isopropanol supplemented with 15 mM ammonium formate. The eluates were centrifuged for 10 min at 20,000 g before vacuum-drying and reconstitution in 100 µL 50% ACN each. 1 µL was used for LC–MS analysis.

#### Proteomics

Proteomics analysis was conducted starting with 100 mg of frozen mycelium sample which were weighed into glass bead-milling tubes and 1 mL of reducing and alkylating buffer (100 mM Tris–HCl; pH = 8.5, 1% sodium dodecyl sulphate, 10 mM tris(2-carboxyethyl) phosphine, 40 mM 2-chloroacetamide) was added. Lysis was conducted by bead-milling (2 min, 6 m s^−1^) and subsequent sonication (10 s, 10% intensity). The lysed samples were spun down for 12 min at 20,000 g and 20 °C and the supernatant was transferred into fresh Eppendorf tubes. The supernatants were heated to 95 °C for 10 min at 330 rpm to perform reduction and alkylation of the proteins. 100 μg of protein per sample (according to reducing agent compatible bicinchoninic acid assay protein estimation (Thermo Fisher Scientific)) were subjected to acetone precipitation by adding NaCl to a final concentration of 10 mM and incubation for 5 min at room temperature. Subsequently, 4 × volumes of acetone were added and samples were incubated for 2 min. After centrifugation (15 min at 15,000*g*) the supernatant was removed. Dried samples were dissolved in 25% trifluoroethanol in 100 mM Tris–HCl (pH = 8.5) and subjected to vortexing and sonication until completely dissolved. For protein digest, samples were diluted to 10% trifluoroethanol using 100 mM ammonium bicarbonate. Trypsin (Promega, Fitchburg, WI, USA) was added in a 1:50 enzyme to protein ratio and digest was performed overnight at 37 °C and 500 rpm. The following day, 500 ng of digested sample were loaded on Evotips, according to the protocol of the manufacturer (Evosep, Odense, DK).

#### Scaled-up extraction for NMR analysis

Around 5 g of mycelium of both OETriliR and ΔTriliE strains were extracted by first grinding in liquid nitrogen to a fine powder and subsequently bead-milling in diethyl ether. After removing cell debris and beads, the diethyl ether extracts were washed two times with ddH_2_O, dried under a nitrogen stream, and reconstituted in 60% ACN in 15 mM ammonium formate for subsequent HPLC separation and fractionation.

### HPLC(–MS/MS) analysis

#### Metabolomics of mycelium

After 1:10 dilution of the polar extracts, ilicicolin H (C_27_H_31_NO_4_) was measured employing an untargeted metabolomics workflow in positive polarization mode on a Bruker timsTOF Pro equipped with a VIP-HESI source (Bruker Corporation, Billerica, MA, USA). The frontend was a Thermo Fisher Scientific Vanquish H UHPLC with a Waters Acquity BEH C18 column (150 mm × 1 mm ID, 1.7 μm; Waters Corporation, Milford, MA, USA). Mobile phase A was 60% acetonitrile in 15 mM ammonium formate; mobile phase B was 10% acetonitrile in isopropanol supplemented with 15 mM ammonium formate. The following gradient was employed at 50 °C: 0 min, 2% B, 100 µL min^−1^; 15 min, 100% B; 70 µL min^−1^; 22 min, 100% B, 70 µL min^−1^, followed by 5 min re-equilibration to starting conditions (2% B, 100 µL min^−1^). The timsTOF Pro mass spectrometer was operated in positive ionization mode with enabled trapped ion mobility spectrometry (TIMS) at 100% duty cycle (100 ms ramp time). Source capillary voltage was set to 4500 V and dry gas flow to 8 L min^−1^ at 230 °C. Sheath Gas Flow was set to 4.0 L min^−1^ at a temperature of 100 °C, with active exhaust being activated. Scan mode was set to parallel accumulation serial fragmentation (PASEF) with a scan range from 300 to 1,300 m*/z*, using a mobility (1/K0) window from 0.8 to 1.81 V*s cm^−2^. Collision Energy was set to 45 eV.

To obtain fragmentation spectra for all features without pre-filtering by ion mobility, measurements for molecular networking were conducted without trapped ion mobility spectrometry (TIMS off). The LC eluents and gradient remained unaltered. The timsTOF Pro mass spectrometer was operated in positive ionization mode, with the same settings as described above. Only the Sheath Gas Flow temperature was changed to 200 °C. Scan mode was set to Auto MS/MS with 12 Hz MS spectra rate and 16 Hz MS/MS spectra rate, resulting in a total cycle time of 0.5 s. Scan range was set from 20 to 1,300 m*/z*. The mass spectrometry metabolomics and molecular networking data files have been deposited to the MassIVE repository with the dataset identifier MSV000095813.

#### Metabolomics of medium

HPLC–MS/MS measurement of the prepared standards and extracts for the quantification of ilicicolin H in the medium was carried out as described in the methods Sect. "Metabolomics of mycelium".

#### Proteomics

Starting with 500 ng digest, peptides were separated on the Evosep One equipped with an Ionopticks Aurora Series UHPLC C18 column (15 cm × 75 µm ID, 1.7 µm; Ionopticks, Fitzroy, VIC, Australia). The LC–method Whisper_40SPD was used with solvent A being 0.1% formic acid in water and solvent B acetonitrile containing 0.1% formic acid while maintaining the column at 40 °C. The timsTOF HT mass spectrometer (Bruker Daltonics, Bremen, Germany) was operated in positive mode with enabled trapped ion mobility spectrometry (TIMS) at 100% duty cycle (100 ms ramp time). Source capillary voltage was set to 1500 V and dry gas flow to 3 L min^−1^ at 180 °C. Scan mode was set to data-independent parallel accumulation–serial fragmentation (diaPASEF) using parameters previously optimized with py_diAID [26]. In brief, 24 isolation windows from *m/z* 300 to 1,200 and 1/K0 0.7 to 1.35 V*s cm^−2^ were defined. After MS1 scan, 2 isolation windows were fragmented per TIMS ramp resulting in an overall DIA cycle time of 1.38 s. The mass spectrometry proteomics data and DIA windows details have been deposited to the ProteomeXchange Consortium via the PRIDE [27] partner repository with the dataset identifier PXD055742.

#### Scaled-up extraction for NMR analysis

Separation and fractionation of the mycelial diethyl ether extracts was conducted on a Thermo Fisher Scientific Vanquish F UHPLC with a Waters XBridge BEH C18 column (150 mm × 4.6 mm ID, 2.5 μm; Waters Corporation, Milford, MA, USA). Mobile phase A was 60% acetonitrile in 15 mM ammonium formate; mobile phase B was 10% acetonitrile in isopropanol supplemented with 15 mM ammonium formate. The following gradient was employed at 50 °C and a flow-rate of 1 mL min^−1^, for OETriliR samples: 0 min, 2% B; 10 min, 45% B; 11 min, 98% B; 15 min, 98% B, followed by 5 min re-equilibration to starting conditions (2% B, 1 mL min^−1^). UV-absorption was detected at a wavelength of λ = 320 nm. For ΔTriliE samples, the gradient was slightly adapted for better isomer separation: 0 min, 2% B; 7 min, 33.5% B; 10 min, 40% B; 11 min, 98% B; 15 min, 98% B, followed by 5 min re-equilibration to starting conditions. Flow-rate and separation temperature remained unchanged (1 mL min^−1^, 50 °C). Fractions were manually collected, united, and dried under vacuum. Dried extracts were quantified gravimetrically. 6.7 mg ilicicolin H were extracted from 5 g dried OETriliR mycelium and employed for NMR analysis. This corresponds to 1.34 mg ilicicolin H per gram of dried OETriliR mycelium (= 1.34 µg per mg mycelium).

To purify ilicicolin H and K for the microbial tests, diethyl ether extracts from OETriliR mycelia were fractionated on an Autopurification system (Waters Corporation, Milford, MA, USA) using an ACQUITY QDa Detector in combination with a 2998 Photodiode Array Detector, equipped with a XSELECT CSH C18 OBD Prep column (150 mm × 30 mm ID, 5 μm; Waters Corporation, Milford, MA, USA). The following gradient was employed at room temperature (25 °C) and a flow-rate of 20 mL min^−1^: 1 min, 2% B; 21 min, 45% B; 23.40 min, 98% B; 36 min, 98% B; 37 min, 2% B, followed by 13 min re-equilibration to starting conditions (2% B, 20 mL min^−1^). The PDA detector was set to a wavelength range from λ = 200 nm to λ = 450 nm, with a resolution of 1.2 nm and a sampling rate of 10 points s^−1^.

### NMR analysis

All samples were measured on a Bruker Avance III 600 MHz spectrometer with Prodigy nitrogen cryo BBFO{H-F} inverse probe head. Spectra were recorded at 298 K and are referenced to the residual solvent signal.

### Antifungal activity assays

To determine the broth dilution minimum inhibitory concentration (MIC) of the isolated substances, we conducted microdilution tests following the EUCAST guidelines for yeast (https://www.eucast.org/astoffungi/methodsinantifungalsusceptibilitytesting; EUCAST E.Def 7.4 October 2023 and EUCAST E.DEF 9.4 March 2022). Briefly, ilicicolin H and ilicicolin K were dissolved in DMSO to a concentration of 20.0 mg mL^−1^. These stock solutions were then diluted to prepare additional working solutions with concentrations of 12.8, 6.4, 3.2, 1.6, 0.8, 0.4, 0.2, 0.1, and 0.05 for *S. cerevisiae* and 15.0, 10.0, 5.0, 2.0, 1.0, 0.5, 0.2, 0.1, 0.05 and 0.02 mg mL^−1^ for *C. auris*. All solutions were diluted 1:100 in double strength RPMI 2% G supplemented with Tween-20 (RPMI 1640 with L-glutamine without sodium bicarbonate [Thermo Scientific, Catalog number: 31800089], 20.8 g L^−1^; MOPS, 69.06 g L^−1^; glucose, 36 g L^−1^; Tween-20, 0.004% [v/v]; pH adjusted to 7.0 with NaOH). Uracil was added to a final concentration of 40 mg L^−1^ to the double strength RPMI 1640 2% G. A 0.0 mg L^−1^ control was prepared by adding DMSO 1:100 to RPMI 2% G. The *S. cerevisiae* inoculum was prepared by incubating the strain CEN.PK113-5D [28] on YPD agar (20 g L^−1^ peptone, 20 g L^−1^ glucose, 10 g L^−1^ yeast extract) at 30°c for 72 h. The *C. auris* inoculum was prepared by incubating the DSM 21092 strain on potato dextrose agar at 35 °C for 24 h. In both cases, several distinct colonies (≥ 1 mm) were suspended in distilled water, homogenized for 15 s, and the cell density adjusted to λ = 530 nm = 0.1 by adding sterile water. This resulted in a suspension with 1–5 × 10^6^ CFU mL^−1^, equivalent to 0.5 McFarland. The *C. auris* suspension was further diluted 1:10 in distilled water providing a working solution with 1–5 × 10^5^ CFU mL^−1^. Finally, 100 µL of the supplemented media was mixed with 100 µL of inoculum in a well of a sterile, 96-well microdilution plate with flat-bottom wells, resulting in given test concentrations of ilicicolin H and K. The assay was performed in technical triplicates (*S. cerevisiae*) or quadruplicates (*C. auris*), with sterile water instead of inoculum used as a blank. The plates were incubated at 30 °C (*S. cerevisiae*) or 35 °C (*C. auris*) for 24 h and the optical density was measured on a Promega GloMax microplate reader (Promega) at 560 nm (*S. cerevisiae*) or a Tecan INFINITE 200pro plate reader (Tecan Trading AG, Switzerland) at λ = 530 nm (*C. auris*). A growth inhibition of at least 50% compared to the DMSO-supplemented medium was defined as the MIC cut-off.

### Data analysis

#### General utilized software

Marvin was used for drawing, displaying and characterizing chemical structures, substructures and reactions, Marvin 22.6.0, Chemaxon (https://www.chemaxon.com). For fragmentation spectra analysis, SIRIUS [29] version 5.8.6 was used for molecular formula identification and CSI:FingerID [30, 31] for structure database search and substructure annotation. MZmine version 4.0.3 (https://mzio.io/) was used for feature finding and to conduct molecular networking [32–34]. The utilized MZmine batch file is available via the MassIVE repository (MSV000095813), together with the corresponding raw files.

#### Polar extracts and quantification of ilicicolin H in medium

Ilicicolin H (C_27_H_31_NO_4_) was quantified on MS1 level employing the open-source application Skyline version 22.2 [35]. Mass-to-charge, retention time, and CCS were manually matched to an authentic standard. For quantification of ilicicolin H in the medium, an external calibration curve was created, using the areas and known concentrations of the matrix-matched standards (Figure S6).

#### Proteomics

Proteomics data were analyzed using the software DIA-NN 1.8.1 [36, 37]. For *Trichoderma reesei (Hypocrea jecorina*, taxonomy ID 51453, 19,246 entries), all reviewed (Swiss-Prot) and unreviewed (trEMBL) FASTA files were downloaded from UniProtKB (https://www.uniprot.org/) on the 26th of June 2023. The database was manually extended with common contaminants (https://www.thegpm.org/crap/) resulting in 19,366 entries, and used for a library-free search with FDR set to 1%. Deep learning-based spectra, retention time and ion mobility prediction were enabled, minimum fragment ion *m/z* was set to 200 and maximal ion fragment *m/z* to 1800. Trypsin was employed as protease and the maximum number of missed cleavages was configured with 2. Peptide length was adjusted to a range from 7 to 30 amino acids. Cysteine carbamidomethylation was set as a fixed and methionine oxidation as a variable modification, allowing a maximum of one variable modification per peptide. DIA-NN optimized the mass accuracy automatically using the first run in the experiment. This resulted in a list of 6039 proteins with their corresponding LFQ values. The DIA-NN search results are available via ProteomeXchange with the identifier PXD055742.

Data processing using protein group quantities was done with Perseus software version 2.0.11 [38]. Intensities were log2 transformed and contaminants removed. The matrix was then filtered to contain 100% valid values in at least one group. This reduced the matrix to 5977 proteins, and remaining missing values were imputed from a normal distribution (downshift 1.8, width 0.3). Histograms of data distribution per each sample highlighting imputed values are displayed in Figure S7. Principal component analysis was performed without category enrichment. Volcano blots of protein expression differences were generated using the following criteria: S0 of 0.1 and permutation-based FDR set to 5% to correct for multi-testing with 250 randomizations.

## Results

### Activation of the ilicicolin H BGC by overexpression of TriliR.

The ilicicolin H BGC in *T. reesei* QM6a contains, next to the biosynthetic genes *triliA-E*, a gene encoding for a transcription factor (Protein ID 72993, Fig. 1A). The JGI v2.0 gene model [39] suggested a coding region with 1,389 bp encoding for a protein with 462 aa (Supplemental File TriliR_gene_models.fasta) comprising only a partial fungal transcription factor middle homology region (FTFMHR, cd12148 of the conserved domain database [40]) at residues 16–231 with an E-value of 4.82e-15 but no DNA-binding domain [41]. An alternative gene model was suggested for *T. reesei* strain RutC-30 [42] (Protein ID 74475) and the chromosome level genome of QM6a [43] (Protein ID 110738) (Supplemental File TriliR_gene_models.fasta); this model does not contain any introns, consists of 2,364 bp encoding for a protein with 787 aa, and it contains a full-length FTFMHR at residues 270–733 (E-value 1.34e-27) and a GAL4-like zinc cluster DNA-binding domain at residues 11–47 (E-value 9.28e-10). Consequently, we used the longer gene model for our molecular biological work and refer to the gene and protein as *triliR* and TriliR, respectively.

To overexpress TriliR, we put an altered genomic sequence (silent mutation at R227, Supplemental File TriliR_gene_models.fasta) under the control of the constitutive promoter of *tef1* and inserted it in front of the *pyr4* locus into the *T. reesei* strain QM6a Δpyr4 as previously described [19] (Figure S1). We could detect substantially higher transcript levels of all biosynthetic genes in the ilicicolin H BGC in the strain OETriliR (approx. 300 to 1,500 times higher), compared to the control strain (Supplemental File RT-qPCR.xlsx, Figure S4).

### Proteomics and targeted ilicicolin H analysis

**Fig. 2:**
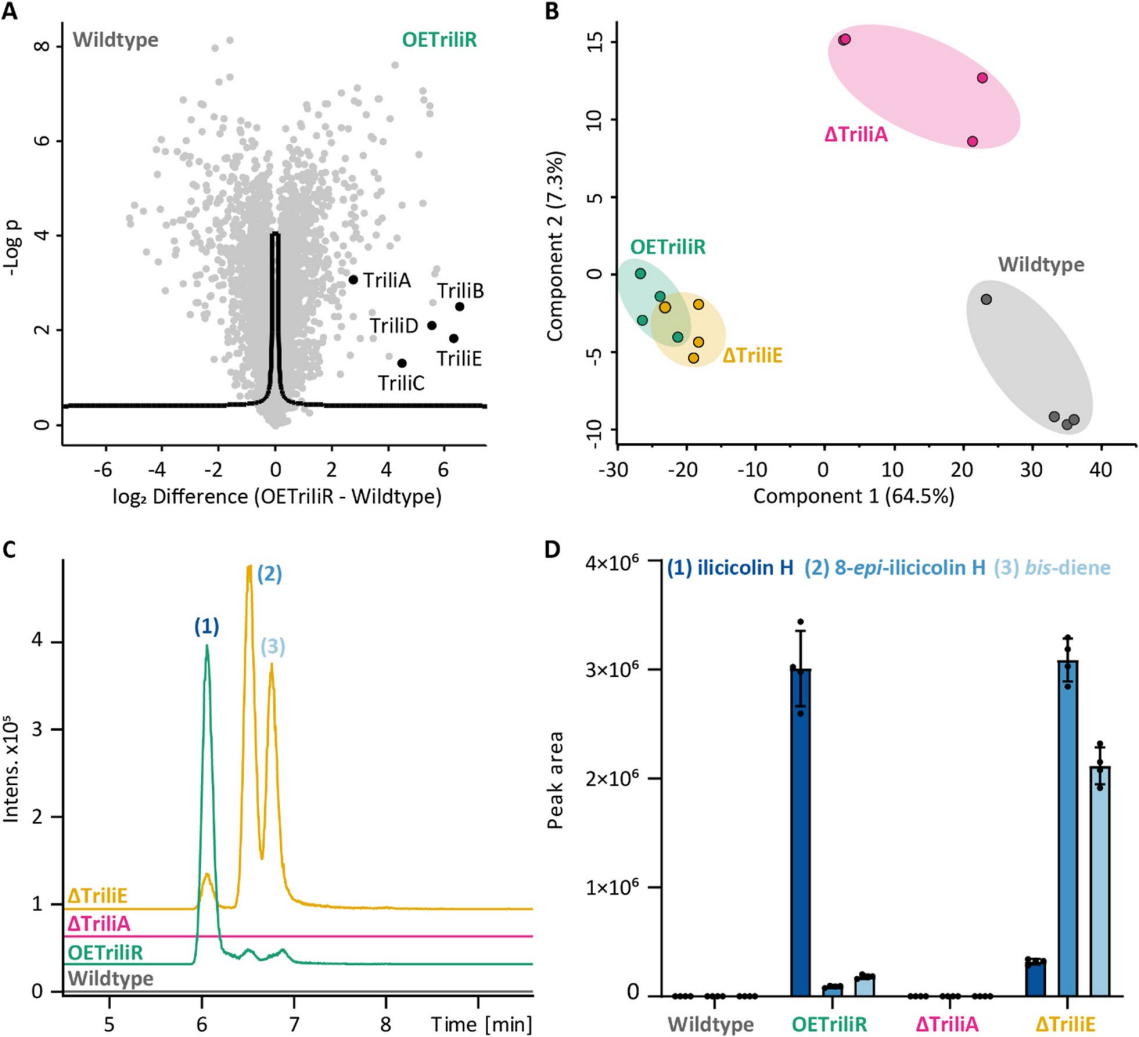
**A** Significant upregulation of all five enzymes involved in ilicicolin H production on protein-level, OE vs. WT (FDR: 0.05, s: 0.1). **B** PCA clustering of the four strains. **C** Extracted ion current chromatograms (EICCs) for ilicicolin H (C_27_H_31_NO_4_, [M + H].^+^ 434.2326 ± 0.02) of the four utilized strains. Peak (1**)** represents ilicicolin H, **(**2**)** 8-*epi*-ilicicolin H and **(**3**)** the *bis*-diene.** D** MS1 peak areas of ilicicolin compounds (n = 4)

This successful overexpression on mRNA level was also confirmed on proteomic level, with all five enzymes TriliA-E being significantly upregulated in the OETriliR strain compared to the wildtype strain**,** as depicted in Fig. 2A. Principal component analysis (PCA, Fig. 2B) revealed that the conducted genetic modifications resulted in the emergence of three distinct clusters. Specifically, sole overexpression of the ilicicolin H cluster (strain designation: OETriliR) leads to a marked change in the protein expression profile, evident from its clustering distant to the wildtype samples. Samples with ilicicolin-BGC overexpression and simultaneous knockout of the epimerase *triliE* (strain designation: ΔTriliE), the enzyme catalyzing the last step of ilicicolin H formation, demonstrate an overlap with the OETriliR cluster, suggesting minimal proteomic changes following the *triliE* knockout. On the contrary, upon ilicicolin-BGC overexpression and simultaneous knockout of *triliA* (strain designation: ΔTriliA), which initiates ilicicolin H formation, a more wildtype like proteome is observed, compared to the other overexpressing strains.

Targeted metabolomics analysis of ilicicolin H revealed that the *T. reesei* wildtype strain does not produce ilicicolin H or direct biochemical precursors in quantities detectable by the employed assay (LOD of ilicicolin H: 21.4 ng mL^−1^, Figure S5) under basal conditions, indicating that the ilicicolin H BGC is indeed silent under common cultivation conditions (Figure S4). Upon overexpression of TriliR (OETriliR), the BGC is successfully activated and ilicicolin H gets produced in high yields, as seen in the extracted ion current chromatograms (EICCs) in Fig. 2C and D. Overall, the vast majority of product can be found in the mycelium, with values of 1.34 µg ilicicolin H per mg mycelium (see Methods Sect. "Scaled-up extraction for NMR analysis") vs. around 6 ng of ilicicolin H in the culture supernatant per mg mycelium (Table S3, factor ~ 225 less). Upon knockout of *triliA* (ΔTriliA), the enzyme initiating ilicicolin H biosynthesis, the production of ilicicolin H is completely halted, as depicted in Fig. 2C. This confirms that TriliA is necessary to initiate the formation process.

### Ilicicolin H isomers in detail

Extracted ion current chromatograms of ilicicolin H reveal three isomers at the expected *m/z* value (Fig. 2C), with peak **(**1**)** representing the main product, whereas peak (2**)** represents 8-*epi*-ilicicolin H and peak **(**3) the *bis*-diene, respectively. The epimerase TriliE catalyzes the last step in the ilicicolin H biosynthesis, namely the epimerization from 8-*epi*-ilicicolin H **(**2**)** to ilicicolin H (1**)** [12]. **(**2**)** in turn is a product of a putative S-adenosyl-methionine (SAM)-dependent Diels-Alderase [14], that catalyzes the transformation from *bis*-diene **(**3**)** to 8-*epi*-ilicicolin H **(**2**)**. Knockout of the epimerase-encoding gene *triliE* leads to a strong decrease in the production of **(**1**)**, and instead, (2**)** and **(**3**)** increase drastically in their abundance (Fig. 2C and D). This is in contrast to the previous report by Zhang *et* *al*. [12], where deletion of the epimerase-encoding gene yielded mainly 8-*epi*-ilicicolin H (2**)** and the biosynthetic precursor of the *bis*-diene, the tetramic acid (4).

The fact that ilicicolin H is still present even if *triliE* is deleted, albeit at a significantly lower level, corroborates the results by Zhang *et* *al.* [12], who showed that nonenzymatic epimerization of **(**2**)** to **(**1**)** can occur, more preferably at higher pH values. However, in the presence of TriliE the epimerization process is substantially improved, underlining the necessity of this enzyme to produce the final product ilicicolin H in high yields in *T. reesei*.

### Molecular networking and discovery of the novel compound ilicicolin K

**Fig. 3:**
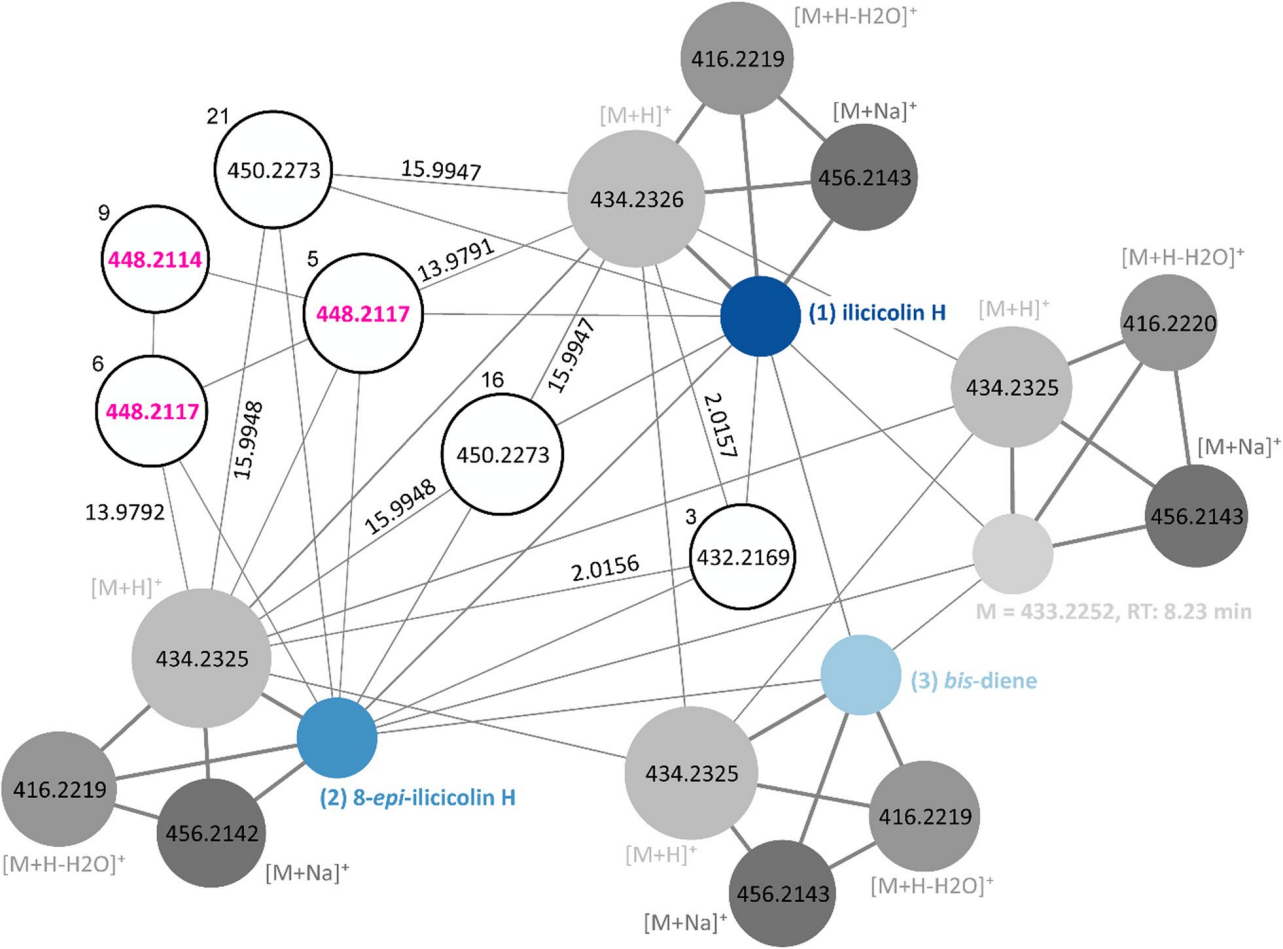
Ion-identity molecular networking using MZmine 4 shows the connections between the previously known ilicicolin H compounds (1), **(**2**) **and (3) and reveals the presence of several related compounds. Size of the nodes corresponds to the log10_sum_intensity of the features, width of the edges to the similarity score. Dark grey colors indicate ionization adducts ([M + H]^+^, [M + Na]^+^, [M + H-H_2_O]^+^). White nodes with black contour represent unknown features, the numbers next to them indicate their feature ID within the network

Molecular networking of the untargeted metabolomics data using MZmine 4 combined several features to an “ilicicolin” network (Fig. 3). In addition to ilicicolin H **(**1**)** and its pathway intermediates (2**)** and **(**3**)**, a fourth compound with a monoisotopic mass of 433.2252 Da was detected. This compound, like **(**1**)**, **(**2**)** and **(**3**)**, exhibited the same ionization adducts ([M + H]^+^, [M + H-H_2_O]^+^, [M + Na]^+^) but eluted substantially later than the other three. Despite similar fragmentation spectra and *m/z* ratio, this feature is unlikely to be ilicicolin H or one of its measured intermediate products because of a vastly different retention time (RT = 8.23 min vs 5.84 min for **(**1**)**, 6.31 min for (2**)** and 6.57 min for **(**3**)** and the feature not being visible when ion mobility is turned on in the applied mobility (1/K0) window from 0.8 to 1.81 V*s cm^−2^.

To the network nodes of **(1)** and **(2)**, six additional features were clustered with *m/z* values of 432.2169 (feature ID 3), 450.2273 (IDs 16, 21), and 448.2117 (IDs 5, 6, 9). ID 9 with an *m/z* value of 448.2114 was matched to the other two 448.2117 *m/z* nodes (IDs 5 and 6) only. Manual inspection revealed that this feature was an artifact of the feature detection, rendering it redundant.

**Fig. 4:**
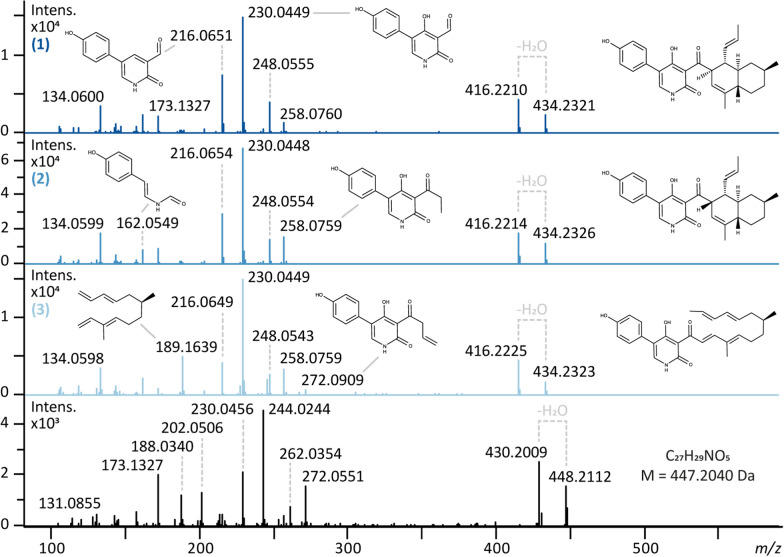
Fragmentation spectra of ilicicolin H (1**)**, 8-*epi*-ilicicolin H **(**2**)**, the *bis*-diene (3**)** and the novel ilicicolin compound with the proposed sum formula C_27_H_29_NO_5_. Substructure annotation was conducted using SIRIUS

Molecular formula prediction by SIRIUS revealed for 432.2169 *m/z* (ID 3) the sum formula C_27_H_29_NO_4_, which could correspond to the compound ilicicolin J (Fig. 1C). For 450.2273 *m/z* (IDs 16 and 21), C_27_H_31_NO_5_ was proposed, implying an oxidized ilicicolin H variant. For 448.2117 *m/z* (IDs 5, 6 & 9; highlighted in pink), C_27_H_29_NO_5_ was suggested, which corresponds to a neutral mass of 447.2040 Da. This unusual modification—apparently corresponding to an oxidation with simultaneous loss of two hydrogen atoms—caught our attention and we conducted further investigation by comparing its fragment spectra to those of (1**)**, (2**)** and **(**3**)** in Fig. 4.

Although **(**1**)**, **(**2) and **(**3**)** are isomeric compounds with the sum formula C_27_H_31_NO_4_ and an exact mass of 433.2253 Da, their fragmentation spectra show certain differences (Fig. 4). Whereas **(**1**)** and **(**2**)**, the two epimers, reveal nearly identical fragments with very similar intensities, (3**)** differs in regard to fragments which are part of the open chain (decalin unit) of the molecule. This becomes apparent by an intense fragment at 189.1639 *m/z*, which is far less pronounced in **(**1) and **(**2**)**, and the fragment at 272.0909 *m/z*. The first one constitutes the fragmented decalin unit, which fragments more readily in open conformation than when having undergone the Diels–Alder cycloaddition. The second one is the phenyl-pyridone unit with a part of the decalin unit attached. Overall, all three compounds fragment preferably directly between these two moieties, resulting in the most pronounced ion with an *m/z* value of 230.0449. The novel compound shows a characteristic shift of + 13.98 Da for many of the fragments, compared to the spectrum of **(**1**)**, suggesting a somewhat fragmentation resistant modification. While both compound (3) and the novel compound display a mass fragment around 272 *m/z*, these fragments correspond to distinct molecular formulas. The signal at 272.055 *m/z* in the novel compound’s spectrum arises from the modified fragment at 258.0759 *m/z* (observed in compounds **(**1**)**, **(**2**)**, and **(**3)), incorporating the previously described mass shift of + 13.98 Da, resulting in a theoretical *m/z* value of 272.0559 (observed: 272.0551 *m/z*). Judging from the location of the shift, the modification is most likely located in the phenyl-pyridone moiety of the molecule.

**Fig. 5:**
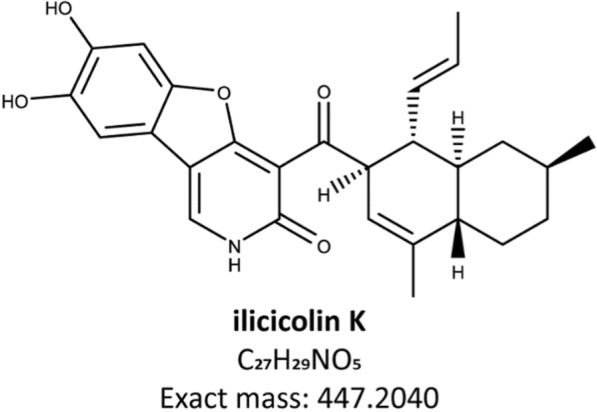
The newly discovered compound ilicicolin K

In accordance with these findings, NMR analysis revealed that the compound is actually an ilicicolin H derivative which was hydroxylated at the tyrosine moiety of the molecule with simultaneous ring formation of the hydroxyl group at the pyridone towards the tyrosine, yielding a dihydroxy-dihydro-oxa-azafluorene (Fig. 5). We termed this novel compound ilicicolin K.

Because of the structural similarities, we next tested whether ilicicolin K shares the antifungal characteristics of its biosynthetic relatives. Similar to ilicicolin H, ilicicolin K exhibits antifungal activity against *S. cerevisiae* and the potential human pathogen strain *Candida auris*. The MIC of ilicicolin K against *S. cerevisiae* was approximately 4 µg mL^−1^ and against *C. auris* 39.4 µg mL^−1^, compared to > 50 µg mL^−1^ and 0.77 µg mL^−1^ for ilicicolin H, respectively (Figure S8).

## Discussion

Although the ilicicolin H BGC has been previously described, earlier studies exclusively relied on heterologous expression systems to elucidate its pathway. In this study, we present the successful genetic activation of the typically silent, albeit functional, ilicicolin H BGC of *T.* *reesei* in its native host. Recently, another successful activation of the ilicicolin BGC in *T.* *reesei* was reported by Wang *et al*. [44]. Whereas the authors exchanged the promoters of TriliA-E resulting in the production of ilicicolin H, we achieved activation by overexpressing the cluster's transcription factor, TriliR, under the control of the constitutive *tef1* promoter. Our results demonstrate successful expression of the cluster's enzymes at both mRNA and protein levels. Furthermore, targeted metabolomics analysis confirmed high-yield production of ilicicolin H upon overexpression of TriliR.

In this study, the transcription factor TriliR was overexpressed, ensuring that the resulting expression levels of the enzymes TriliA–TriliE maintained their natural ratios relative to each other, mirroring normal physiological activation conditions. This approach allows for a precise assessment of each enzyme's role within the cluster. In contrast, investigation of enzymatic functions in heterologous expression systems poses the risk of producing biased results because host enzymes can promote or interfere with the artificial pathway, potentially leading to the formation of undesired compounds or shunt products that would not naturally occur in the native host.

This potential discrepancy becomes apparent when comparing our findings to those of Shenouda *et* *al.* [14] who investigated the same BGC from *T. reesei* through heterologous expression in *A. oryzae.* The authors registered high amounts of ilicicolin H even in the absence of TriliE, suggesting that TriliE is not essential for ilicicolin H formation. This observation is in contrast to the results from this study employing a homologous expression system, where the absence of TriliE led to a drastic reduction in ilicicolin H levels. Additionally, Shenouda *et* *al.* [14] detected several acetylated shunt products that we—in the homologous system—could neither observe in the corresponding extracted ion current chromatograms (EICCs), nor did they appear in the molecular network search. Further dissimilarities were observed in the accumulated intermediates upon TriliE knockout. Whereas Zhang *et* *al.* [12] reported an increase in 8-*epi*-ilicicolin and tetramic acid in their system lacking the epimerase, we observed accumulation of the *bis*-diene intermediate next to 8-*epi*-ilicicolin. A possible explanation for that could be that the ring-expanding cytochrome P450 (IccC [12], TriliC [14]), responsible for the conversion of the initially formed tetramic acid to the *bis*-diene [12], is more efficient in the native *Trichoderma reesei* host system. This might either be caused by higher expression in the engineered BGC or, perhaps more likely, by a better cofactor supply by a matched P450 reductase [45]. This illustrates how varying hosts can influence product formation in different ways.

By applying molecular networking to untargeted metabolomics data, we identified several features related to ilicicolin H and its pathway intermediates. NMR analysis of one of these compounds, which exhibited an unusual modification, confirmed a novel ilicicolin product that we have named ilicicolin K. Although numerous attempts of biotransformation and chemical derivatization of ilicicolin H **(**1) have been undertaken already [2, 7, 8], the unique modification observed in ilicicolin K—an intramolecular etherification of the hydroxyl group at the pyridone towards the tyrosine moiety of the molecule—has not yet been reported. This suggests that the formation of this product is not merely a spontaneous side reaction but may be catalyzed by highly specific enzymes.

The antifungal assays demonstrated that ilicicolin K exhibits activity against *S. cerevisiae* and *C. auris*. This is plausible since the β-diketone group of ilicicolin K remained unaltered compared to ilicicolin H** (1)**, which is essential for its activity [7]. For *S. cerevisiae*, we observed a substantially stronger antifungal effect of ilicicolin K compared to ilicicolin H (1) (approx. 4 µg mL^−1^ and > 50 µg mL^−1^, respectively). Previously, Singh *et* *al*. demonstrated that the carbon source in the test medium strongly influences the sensitivity of *S. cerevisiae* against ilicicolin H. On glycerol, a MIC of only 0.012 µg ml^−1^ was achieved, but on glucose, the MIC of ilicicolin H was above 50 µg mL^−1^, matching our results [2]. This can be explained by the mode of action of ilicicolin H, which inhibits the cytochrome bc1 reductase. Yeast can bypass the mitochondrial respiratory chain when cultivated on fermentable carbon sources, such as glucose [46]. In this study, we further observed that ilicicolin K was less potent than ilicicolin H against *C. auris* (39.4 µg mL^−1^ and 0.77 µg mL^−1^). Notably, ilicicolin K reduced the absorbance at higher concentrations more effectively than ilicicolin H. This might be explained by a higher stability of ilicicolin K or a slightly different mode of action. Taken together, our findings suggest that ilicicolin K holds potential as a candidate for future therapeutic strategies targeting fungal infections given that it is strongly antifungal against *S. cerevisiae* on glucose and might be more stable than ilicicolin H.

## Conclusions

In this study, we activated a silent BGC in *Trichoderma reesei*, resulting in the high-yield production of ilicicolin H and the discovery of a previously uncharacterized compound, ilicicolin K. Molecular networking enabled the identification of ilicicolin K, which exhibits stronger antifungal activity against *Saccharomyces cerevisiae* but lower efficacy against *Candida auris*. These findings expand the ilicicolin family and demonstrate the utility of genetic activation for uncovering novel bioactive compounds.

Our work underscores the potential of *T. reesei* as a platform for biosynthetic pathway exploration and antifungal discovery. Future studies should focus on optimizing production yields, further characterizing ilicicolin K's antifungal mechanisms, and exploring its therapeutic applications. This study highlights the promise of microbial biotechnology in discovering and developing new antifungal agents to address pressing challenges in medicine.

## Supplementary Information

Additional file 1

Additional file 2

Additional file 3

Additional file 4

Additional file 5

Additional file 6

## Data Availability

The Supporting information contains information about utilized primers, constructed strains, transcript levels, concentration/amount calculations, proteomics data distribution histograms, curves of the antifungal assay and NMR spectra (Tables S1–S7, Figures S1–S16). Furthermore, the following supplemental files are included: gbk files for Neonectria DH2, T. reesei and T. variabilis; RT-qPCR data and the fasta files of the TriliR gene models. The mass spectrometry metabolomics and molecular networking data files, besides the utilized MZmine batch file, have been deposited to the MassIVE repository with the dataset identifier MSV000095813. The mass spectrometry proteomics data and DIA windows details, besides the DIA-NN search results, have been deposited to the ProteomeXchange Consortium via the PRIDE partner repository with the dataset identifier PXD055742.
