# Supplementary material for: Discovery of the antifungal compound ilicicolin K through genetic activation of the ilicicolin biosynthetic pathway in Trichoderma reesei
Source: Biotechnol Biofuels Bioprod. 2025 Mar 11;18:32. doi: 10.1186/s13068-025-02628-3 (PMC11895301; doi:10.1186/s13068-025-02628-3)
Supplement: Supplementary file 1 — Additional file 1 [file 13068_2025_2628_MOESM1_ESM.pdf]

Supporting Information

Discovery of the antifungal compound ilicicolin K through genetic activation of the ilicicolin biosynthetic pathway in *Trichoderma reesei*

Isabella Burger[1], Matthias Schmal[2], Kathrin Peikert[2], Lukas Fourtis[2], Christoph Suster[3], Christian Stanetty[3], Dominik Schnalzer[3], Barbara Hufnagel[4], Thomas Boettcher[4], Ruth Birner-Gruenberger[1], Robert L Mach[2], Astrid R. Mach-Aigner[2], Matthias Schittmayer[1]*, Christian Zimmermann[2]*

[1] Institute of Chemical Technologies and Analytics, TU Wien, Vienna 1060, Austria

[2] Institute of Chemical, Environmental and Bioscience Engineering, TU Wien, Vienna 1060, Austria

[3] Institute of Applied Synthetic Chemistry, TU Wien, Vienna 1060, Austria

[4] Institute for Biological Chemistry & Centre for Microbiology and Environmental Systems Science, University of Vienna, Vienna, 1090, Austria

*Email: matthias.schittmayer@tuwien.ac.at

*Email: christian.zimmermann@tuwien.ac.at

Table of Contents

Supplementary Tables

Table S1. Utilized primer for cloning, genotyping and RT-qPCR. S3

Table S2. Determined concentrations of ilicicolin H in the culture supernatants of OETriliR and ΔTriliE samples. S4

Table S3. Normalization of determined ilicicolin H amounts in the culture supernatant to the respective amount of fungal mycelium in the flasks. S4

Table S4. Spectroscopic data of **(3)**: shifts of isolated *bis*-diene. S5

Table S5. Spectroscopic data of **(2)**: shifts of isolated 8-*epi*-ilicicolin H. S6

Table S6. Spectroscopic data of **(1)**: shifts of isolated ilicicolin H. S7

Table S7. Spectroscopic data: shifts of isolated and elucidated ilicicolin K. S9

Supplementary Figures

Figure S1. Construction of the strain OETriliR. S10

Figure S2. Construction of the strain ΔTriliA. S11

Figure S3. Construction of the strain ΔTriliE. S12

Figure S4. Transcript level analysis using an RT-qPCR assay. S13

Figure S5. Determination of the limit of detection (LOD) of ilicicolin H using a matrix matched external calibration curve. S14

Figure S6. Matrix matched external calibration curve to quantify ilicicolin H in the medium. S15

Figure S7. Histograms of proteomics data distribution per each sample. S16

Figure S8. Antifungal assays of ilicicolin H **(1)** and the novel ilicicolin K. S17

Figure S9. ^{1}H spectrum (600 MHz) of *bis*-diene **(3)** in acetonitrile-*d*3:DMSO (9:1). S18

Figure S10. ^{13}C spectrum (151 MHz) of *bis*-diene **(3)** in acetonitrile-*d*3:DMSO (9:1). S18

Figure S11. ^{1}H spectrum (600 MHz) of 8-*epi*-ilicicolin H **(2)** in acetonitrile-*d*3. S19

Figure S12. ^{13}C spectrum (151 MHz) of 8-*epi*-ilicicolin H **(2)** in acetonitrile-*d*3. S19

Figure S13. ^{1}H spectrum (600 MHz) of ilicicolin H **(1)** in acetonitrile-*d*3. S20

Figure S14. ^{13}C spectrum (151 MHz) of ilicicolin H **(1)** in acetonitrile-*d*3. S20

Figure S15. ^{1}H spectrum (600 MHz) of ilicicolin K in DMSO-*d*6. S21

Figure S16. ^{13}C spectrum (151 MHz) of ilicicolin K in DMSO-*d*6. S21

References: S22

Supplementary Tables

Table S1. Utilized primer for cloning, genotyping and RT-qPCR.

Name	Sequence	Used for
72993_fwd-AflII	CTTAAGCGCAAGAGCATCCACAAATGG	cloning
72993_MRev_SOE	CAACAAATCTCTCTATCAGCGGGCGCAAGTGCTCGTCTCTC	cloning
72993_MFwd_SOE	GAGAGACGAGCACTTGCGCCCGCTGATAGAGAGATTTGTTG	cloning
72993_rev-SpeI	ACTAGTCTAAACAACCGTGCACCGC	cloning
5_ TriliA _fwd-pRS426	AGTGAGCGCGCGTAATACGACTCACTATAGGGCGAATTGGCAGCACTGTTGGCGATATTC	cloning
5_ TriliA _rev-hph	AGTCACCGGTCACTGTACAGAGCTCGCCTCTAACAATATTCTCTGCTGTG	cloning
hph_fwd-5_ TriliA	CACAGCAGAGAATATTGTTAGAGGCGAGCTCTGTACAGTGACCGGTGACT	cloning
hph_rev-3_ TriliA	GATATCTGCCGTGCTCTCTAATCGCTCGAGTGGAGATGTGGAGTGGGCGC	cloning
3_ TriliA _fwd-hph	GCGCCCACTCCACATCTCCACTCGAGCGATTAGAGAGCACGGCAGATATC	cloning
3_TriliA_rev-pRS426	AGAACTAGTGGATCCCCCGGGCTGCAGGAATTCGATATCACTAAGCGCCACTCTAACAGAAG	cloning
PgpdA_fwd	GAGCTCTGTACAGTGACCGG	Cloning
hph_MR	CGTCAGGACATTGTTGGAGC	cloning / genotyping
hph_MF	GACCTGCCTGAAACCGAAC	cloning / genotyping
TtrpC_rev	AGGTCGAGTGGAGATGTGGAG	cloning
TriliE_5fwd	CAGCCATCATCGCAAACCTAG	cloning
TriliE _5rev-hph	CTCCACATCTCCACTCGACCTGTAATGACCATGCCCCAACC	cloning
TriliE _3fwd-hph	CCGGTCACTGTACAGAGCTCAGGCGTGGTATCTAGGGCAC	cloning
TriliE _3rev	GAGAAGAGAAACGGAGGACTG	cloning
5pyr4_fwd3	CCAGACGGTGATTCACATATACG	genotyping
Ptef_rev-BspTI	CTTAAGTGTGATGTAGCGTGAGAGCTG	genotyping
pyr4_3fwd3	TGCCTTTATCCACATGACGC	genotyping
Tpyr4_rev2	CAGGAAGCTCAGCGTCGAG	genotyping
58285_fwd	GTATGCGTCCTGTGTCTCAC	genotyping
58285_rev	CTGTTACGCAAAGGTCTCCG	genotyping
iliE_5'_genomic	AGGTAGTGAAGTCTCGGTTG	genotyping
iliE_3'_genomic	CTGAGCTCTGGTATGTGGTT	genotyping
sar1fw	TGGATCGTCAACTGGTTCTACGA	RT-qPCR
sar1rev	GCATGTGTAGCAACGTGGTCTTT	RT-qPCR
act1f	TGAGAGCGGTGGTATCCACG	RT-qPCR
act1r	GGTACCACCAGACATGACAATGTTG	RT-qPCR
58285_q1f	GCTGAACATGATGACCAAAGAG	RT-qPCR

58285_q1r	TTCGTTTTCAGGGAGTAGATGC	RT-qPCR
58289_q1f	AGCAGAAGCCTGGTTTGACTAC	RT-qPCR
58289_q1r	GCTTTGCCGAACTGTGTAGG	RT-qPCR
58953_q1f	CGTCAGAGATGAGGAGGAACTG	RT-qPCR
58953_q1r	GGAATCGTTGAGGCAAGCAC	RT-qPCR
Rut75073_q1f	TTGTCAAAGGAGATGATGTCG	RT-qPCR
Rut75073_q1r	TACTACAACGTCGACGAAACC	RT-qPCR
76204_q1f	CCGGAAATTCTCGTTGATAGG	RT-qPCR
76204_q1r	ATTGAGCAGGCTCCATTTGTC	RT-qPCR
72993_q1f	CGTTAACGGCTCTTCGGATAC	RT-qPCR
72993_q1r	GCAGAAAAGGAACTCGCGTG	RT-qPCR

Table S2. Determined concentrations of ilicicolin H in the culture supernatants of OETriliR and ΔTriliE samples. *: significant outlier according to Grubbs' test, significance level: 0.05 (two-sided), Z-value: 1.48919.

	**c [ng µL^{-1}]**	**$\overline{X}$ (c, n = 4) [ng µL^{-1}]**	**± s (c, n = 4) [ng µL^{-1}]**
**OETriliR #1**	13.87	25.3, (15.21 excl. outlier)	20.32 (2.98 excl. outlier)
**OETriliR #2**	55.55*		
**OETriliR #3**	13.13		
**OETriliR #4**	18.63		
**ΔTriliE #1**	4.25	3.13	1.23
**ΔTriliE #2**	3.92		
**ΔTriliE #3**	2.81		
**ΔTriliE #4**	1.53		

Table S3. Normalization of determined ilicicolin H amounts in the culture supernatant to the respective amounts of fungal mycelium in the flasks. *: significant outlier according to Grubbs' test, significance level: 0.05 (two-sided), Z-value: 1.49909.

	**Mycelium amount [mg]**	**Determined concentration of iliH in the medium [ng µL^{-1}]**	**Absolute amount of ilicicolin H in medium [ng]**	**[ng] iliH in medium per [mg] mycelium**
**OETriliR #1**	208.4	13.87	1387.22	6.66
**OETriliR #2**	234.3	55.55	5555.31	23.71*
**OETriliR #3**	192.3	13.13	1312.78	6.83
**OETriliR #4**	304.1	18.63	1862.53	6.13
**ΔTriliE #1**	187.2	4.25	425.10	2.27
**ΔTriliE #2**	241.2	3.92	391.52	1.62
**ΔTriliE #3**	219.6	2.81	280.65	1.28
**ΔTriliE #4**	180.2	1.53	152.90	0.85

Table S4. Spectroscopic data of **(3)**: shifts of isolated *bis*-diene.

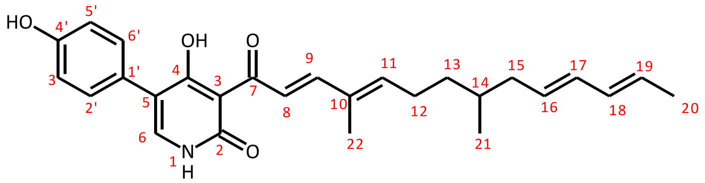

bis-diene **(3)**

POS	Reported (in CDCl3:Acetone 3:1)[2] δ_C	δ_H(MULT.,INT, *J*)	Found (in ACN:DMSO) δ_C	δ_H(MULT.,INT, *J*)
2	162.9	-	162.9	-
3	106.7	-	106.9	-
4	178.0	-	178.2	-
5	114.8	-	114.1[c]	-
6	139.1	7.44 (s, 1H)	140.6	7.41 (s, 1H)
7	194.6		194.8	
8	123.3	8.01 (d, 1H, *J* = 15.5 Hz)	123.7	8.03 (d, 1H, *J* = 15.46)
9	150.1	7.53 (d, 1H, *J* = 15.5 Hz)	159.8	7.53 (d, 1H, *J* = 15.4 Hz)
10	134.5		134.5	
11	144.8	6.01 (t, 1H, *J* = 7.5 Hz)	145.5	6.11 (t, 1H, *J* = 7.27 Hz)
12	26.8	2.22 (m, 2H)	26.9	2.27 (m, 2H)
13	35.5	Ha 1.22 (m, 1H)	35.8	Ha 1.27[b]
		Hb 1.42 (m, 1H)		Hb 1.43 (m, 1H)
14	33.1	1.50 (m, 1H)	33.2	1.53 (m, 1H)
15	40.0	Ha 1.88 (q, 1H, *J* = 13.0, 7.0 Hz)	39.9[d]	Ha 1.90[a]
		Hb 2.02 (q, 1H, *J* = 13.0, 6.0 Hz)		Hb 2.05[b]
16	130.0	5.45 (m, 1H)	130.7	5.55 (m, 1H)
17	131.4	5.88 (m, 1H)	132.17 or 132.20	6.00 (m, 1H)
18	131.6	5.94 (m, 1H)	132.17 or 132.20	6.00 (m, 1H)
19	126.7	5.50 (m, 1H)	127.3	5.55 (m, 1H)
20	17.9	1.65 (d, 3H, *J* = 6.5 Hz)	17.7	1.69 (d, 3H, *J* = 6.9 Hz)
21	19.4	0.85 (d, 3H, *J* = 7.0 Hz)	19.3	0.88 (d, 3H, *J* = 6.7 Hz)
22	12.4	1.84 (s, 3H)	12.2	1.85 (d, 3H, *J* = 1.1)
1’	124.4		124.6	
2’, 6’	130.3	7.26 (d, 1H, *J* = 8.5 Hz)	130.8	7.25 (d, 1H, *J* = 8.6 Hz)
3’, 5’	115.4	6.83 (d, 1H, *J* = 8.5 Hz)	115.6	6.81 (d, 1H, *J* = 8.6 Hz)
4’	156.9		157.7	

[a] Overlayed by ACN solvent signal
[b] Overlayed by Fatty-acid Signal
[c] Hardly visible in 13C-NMR – HMBC Correlation proves position and nature
[d] Overlayed by DMSO Signal – Position from HSQC Correlation

Table S5. Spectroscopic data of **(2)**: shifts of isolated 8-*epi*-ilicicolin H.

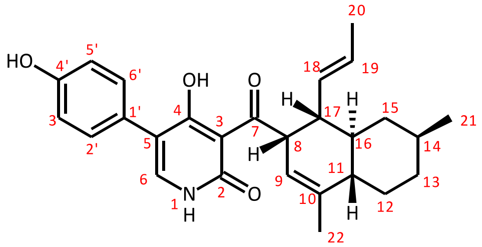

epi-Ilicicolin H **(2)**

POS	Reported (in acetonitrile-*d*3)[2] δ_C	δ_H(MULT.,INT, *J*)	Found (in acetonitrile-*d*3) δ_C	δ_H(MULT.,INT, *J*)
2	162.9	-	162.45	-
3	108.0	-	108.0	-
4	177.6	-	177.8	-
5	114.8	-	114.5	-
6	140.8	7.42 (s, 1H)	140.8	7.40 (s, 1H)
7	209.8	-	209.9	-
8	50.8	5.07 (m, 1H)	50.7	5.07 (m, 1H)[a]
9	120.1	5.47(m, 1H)	120.1	5.45 (m, 1H)
10	141.2	-	141.2	-
11	46.0	1.54 (m, 1H)	46.0	1.56 (m, 1H)[b]
12	30.4	Ha 1.00 (m, 1H)<br>Hb 2.03 (m, 1H)	30.5	Ha 0.98 (m, 1H)<br>Hb 2.06 (m, 1H)
13	36.3	Ha 0.93 (m, 1H)<br>Hb 1.77 (m, 1H)	36.4	Ha 0.98 (m, 1H)<br>Hb 1.77 (m, 1H)
14	33.7	1.41 (m, 1H)	33.7	1.42 (m, 1H)
15	40.7	Ha, 0.44 (q, 1H *J* =24.5 12.0 Hz)<br>Hb 1.74 (m, 1H)	40.7	Ha, 0.46 (app. q, 1H *J* =11.8 Hz)<br>Hb 1.77 (m, 1H)
16	40.6	1.93 (m, 1H)	40.66	1.94 (m, 1H)[c]
17	47.4	2.17 (m, 1H)	47.4	2.18 (m, 1H)
18	133.4	5.45 (m, 1H)	133.5	5.45 (m, 1H)
19	127.3	5.44 (m, 1H)	127.3	5.45 (m, 1H)
20	18.1	1.54 (d, 3H)	18.1	1.56 (d, 3H, *J* = 4.58 Hz)
21	23.0	0.88 (d, 3H, *J* = 6.5 Hz)	23.03	0.90 (d, 3H, *J* = 6.6 Hz)
22	21.5	1.66 (s, 3H)	21.5	1.68 (d, 3H, *J* = 1.6 Hz)
1’	125.4	-	125.5	-
2’, 6’	131.4	7.26 (d, 1H, *J* = 9.0 Hz)	131.5	7.26 (d, 1H, *J* = 8.6 Hz)
3’, 5’	116.0	6.83 (d, 1H, *J* = 9.0 Hz)	116.0	6.83 (d, 1H, *J* = 8.6 Hz)
4’	157.5	-	157.5	-

[a] Peakshape identical to Literature, small coupling between H8 – H17 indicates cis configuration.
[b] Peak overlayed by H20 – shift from HSQC
[c] Peak overlayed by residual solvent signal – shift from HSQC

Table S6. Spectroscopic data of **(1)**: shifts of isolated ilicicolin H.

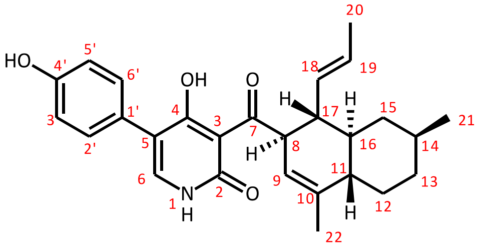

Ilicicolin H **(1)**

POS	Reported (in acetonitrile-*d*3)[2] δC	δH(MULT.,INT, *J*)	Found (in acetonitrile-*d*3) δC	δH(MULT.,INT, *J*)
2	162.8	-	162.9	-
3	107.8	-	107.8	-
4	177.6	-	177.5	-
5	114.6	-	114.3	-
6	140.9	7.43 (s,1H)	141.2	7.41 (s, 1H)
7	210.7	-	210.7	-
8	53.8	4.98 (m, 1H)	53.7	4.98 (m, 1H)[a]
9	120.4	5.21 (m, 1H)	120.6	5.21 (m, 1H)
10	139.1	-	139.0	-
11	45.1	1.67 (m, 1H)	45.2	1.69 (1H, m)
12	30.6	Ha 0.97 (m, 1H)<br>Hb 2.03 (m, 1H)	30.6	Ha 0.98 (1H, m)<br>Hb 2.05 (1H, m)
13	36.2	Ha 0.97 (m, 1H)<br>Hb 1.76 (m, 1H)	36.2	Ha 0.98 (1H, m)<br>Hb 1.78 (1H, m)
14	33.4	1.38 (m, 1H)	33.4	1.39 (m, 1H)
15	40.7	Ha 0.57 (q, 1H)<br>Hb 1.76 (m, 1H)	40.4	Ha 0.58 (dt, 1H, *J* = 13.1, 11.7)<br>Hb 1.78 (1H, m)
16	44.2	1.22 (m, 1H)	44.2	1.23 (m, 1H)
17	45.8	2.48 (m, 1H)	45.8	2.49 (td, 1H, *J* = 10.8, 9.1 Hz)
18	134.2	5.21 (m, 1H)	134.4	5.22 (m, 1H)
19	126.8	5.32 (m, 1H)	126.8	5.33 (dq, 1H, *J* = 15.3, 6.3 Hz)
20	18.0	1.52 (dd, 3H, *J* = 6.5, 1.3 Hz)	18.1	1.53 (dd, 3H, *J* = 6.3, 1.6 Hz)
21	23.0	0.88 (d, 3H, *J* = 6.5 Hz)	23.0	0.89 (d, 3H, *J* = 6.6 Hz)
22	21.0	1.62 (s, 3H)	21.1	1.64 (1H, m)
1’	125.3	-	125.0	-
2’, 6’	131.4	7.26 (d, 1H, *J* = 8.6 Hz)	131.3	7.27 (d, 1H, *J* = 8.6 Hz)
3’, 5’	116.0	6.83 (d, 1H, *J* = 8.6 Hz)	116.1	6.84 (d, 1H, *J* = 8.6 Hz)
4’	157.5	-	158.1	-

[a] Peakshape in accordance with literature

Structure elucidation of ilicicolin K was mostly based on established assignments from ilicicolin H **(1)**, and relative changes of the recorded spectra found. Noticeable the aliphatic region of the molecule stayed unaltered according to NMR. Peak shapes as well as shifts remained comparable to **(1)**. The biggest differences were found in the aromatic region, where the former para-substituted phenyl ring exhibited two uncoupled signals with intensity of 1H each. We therefore assumed that the phenyl ring had to be substituted at four positions. One of the two new substitutions can be explained by the addition of a hydroxy group at position C3' . ^{1}H-NMR shows three acidic protons, two of which show HMBC correlations to aromatic carbon signals that can be attributed to two phenolic OHs groups on the aromatic ring. Especially the general shift of all aromatic carbons in ^{13}C-NMR towards highfield is an indication for electron donating effects into the phenyl ring. The other substitution was hypothesized to be a link of C6' to the OH at C4 of **(1)**. A direct proof of this new link via NMR was impossible due to the lack of protons in the relevant region. Advanced NMR techniques to clarify the carbon skeleton (like INADEQUATE or ADEQUATE) were not feasible with the amount of material at hand. However, the resonance structure of C4 in **(1)** gives C4 a partially carbonylic nature, which is seen in the very far downfield shift of that signal (177 ppm). Etherification of the hydroxyl group at C4 would lead to a stabilization the enol, thus a significantly lower chemical shift. In the structure of ilicicolin K one finds a shift of 16 ppm upfield for that specific signal, to 161 ppm. These values are in the range where *para*-hydroxypyridines and their respective ethers are usually found[3]. Therefore, direct link between the aromatic ring and the hydroxy-group of the former C4 of **(1)** is very likely.

The resulting structure fits to the found mass of 447 m/z and is shown below. Assignment of this structure based on ^{1}H, ^{13}C, COSY, HSQC and HMBC spectra was possible and in compliance with expected signal positions for such a structure.

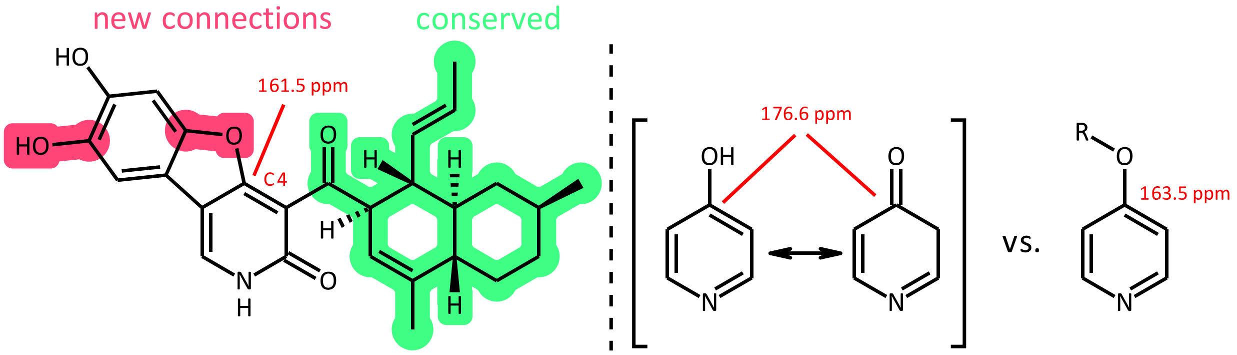

Left: Elucidated structure of the newly found Ilicicolin family member. **Right:** ^{13}C-NMR shift of 4-pyrodinone and the alkylated derivate found in literature[3].

Table S7. Spectroscopic data: shifts of isolated and elucidated ilicicolin K. The numbering for positions in Ilicicolin K was changed compared to Ilicicolin H **(1)**, as the parent system now is fluorene. Since, compared to **(1)**, all carbon positions can be directly mapped, the numbers that would correspond to **(1)** are also indicated.

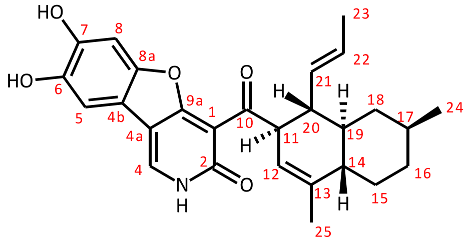

Ilicicolin K

Position in Ilicicolin K	Position in **(1)**	Found (in DMSO-*d6*)[2] δC	δH(MULT.,INT, *J*)	Comparison to **(1)** δC	delta
1	3	110.2	--	107.8	2.4
2	2	165.0	--	162.9	2.1
4	6	131.6	8.30 (s, 1H)	141.2	-9.6
4a	5	110.6	--	114.3	-3.7
4b	1’	111.7	--	125.0	-13.3
5	2’	99.1	6.91 (s, 1H)	131.3	-32.2
6	3’	143.4[a]	--	116.1	27.3
7	4’	146.4[a]	--	158.1	-11.7
8	5’	106.6	7.24 (s, 1H)	116.1	-9.5
8a	6’	150.8	--	131.3	19.5
9a	4	161.5	--	177.5	-16
10	7	201.0	--	210.7	-9.7
11	8	54.2	4.36 (ddt, 1H, *J* = 10.3, 4.1, 2.4 Hz)	53.7	0.5
12	9	120.6	5.24 (m, 1H)	120.6	0
13	10	137.2	--	139.0	-1.83
14	11	44.2	1.60 (m, 1H)	45.2	-1
15	12	29.8	Ha 1.99 (m, 1H)<br>Hb 0.92 (m, 1H)	30.6	-0.8
16	13	35.5	Ha 1.71 (m, 1H)<br>Hb 0.92 (m, 1H)	36.2	-0.7
17	14	32.6	1.34 (m, 1H)	33.4	-0.8
18	15	40.5	Ha 1.71 (m,1H)<br>Hb 051 (app. q, 1H, *J* = 12.1 Hz)	40.4	0.1
19	16	43.5	1.13 (qd, 1H, *J* = 11.5, 2.8 Hz)	44.2	-0.7
20	17	46.0	2.25 (m, 1H)	45.8	0.2
21	18	133.4	5.08 (ddd, 1H, J = 15.2, 9.23, 1.7 Hz)	134.4	-1
22	19	125.9	5.24 (m, 1H)	126.8	-0.9
23	20	18.0	1.28 (dd, 3H, J = 6.4, 1.6 Hz)	18.1	-0.1
24	21	23.1	0.85 (d, 3H, J = 6.6 Hz)	23.0	0.1
25	22	21.2	1.60 (m, 3H)	21.1	0.1
[a] Assignment of C6 and C7 is ambiguous between each other					

Supplementary Figures

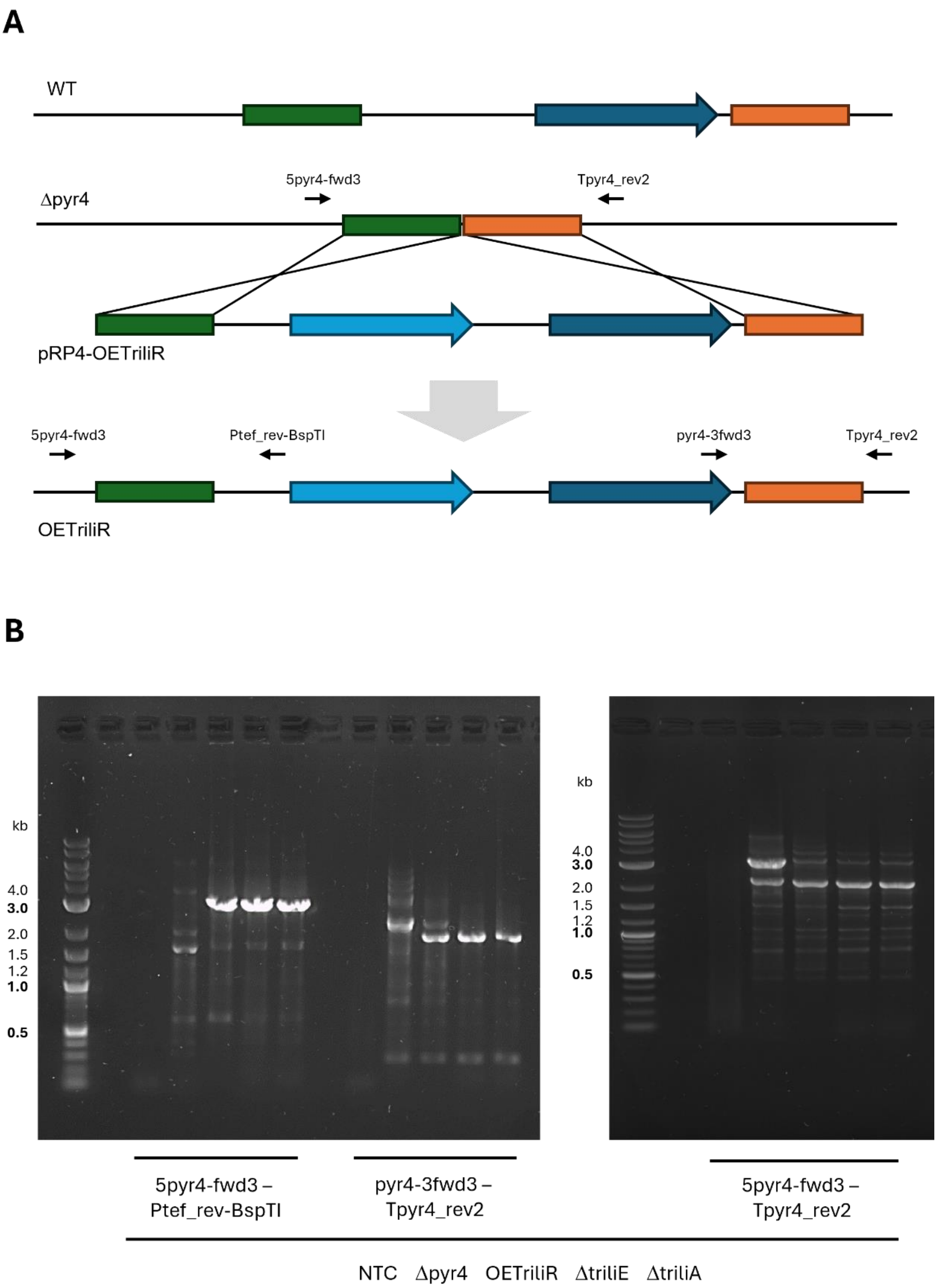

Figure S1. Construction of the strain OETriliR. (A) The coding region for the transcription factor TriliR was put under the control of the *tef1* promoter and inserted into the *pyr4* locus of the recipient strain Δpyr4 using the strategy described by Derntl *et al.*[4]. (B) The insertion of the TriliR expression cassette was tested on a genomic level by suitable PCR assays using the indicated primers and the chromosomal DNA of the used strains. NTC, no template control. 1 kb Plus DNA Ladder (NEB) was used for size comparison.

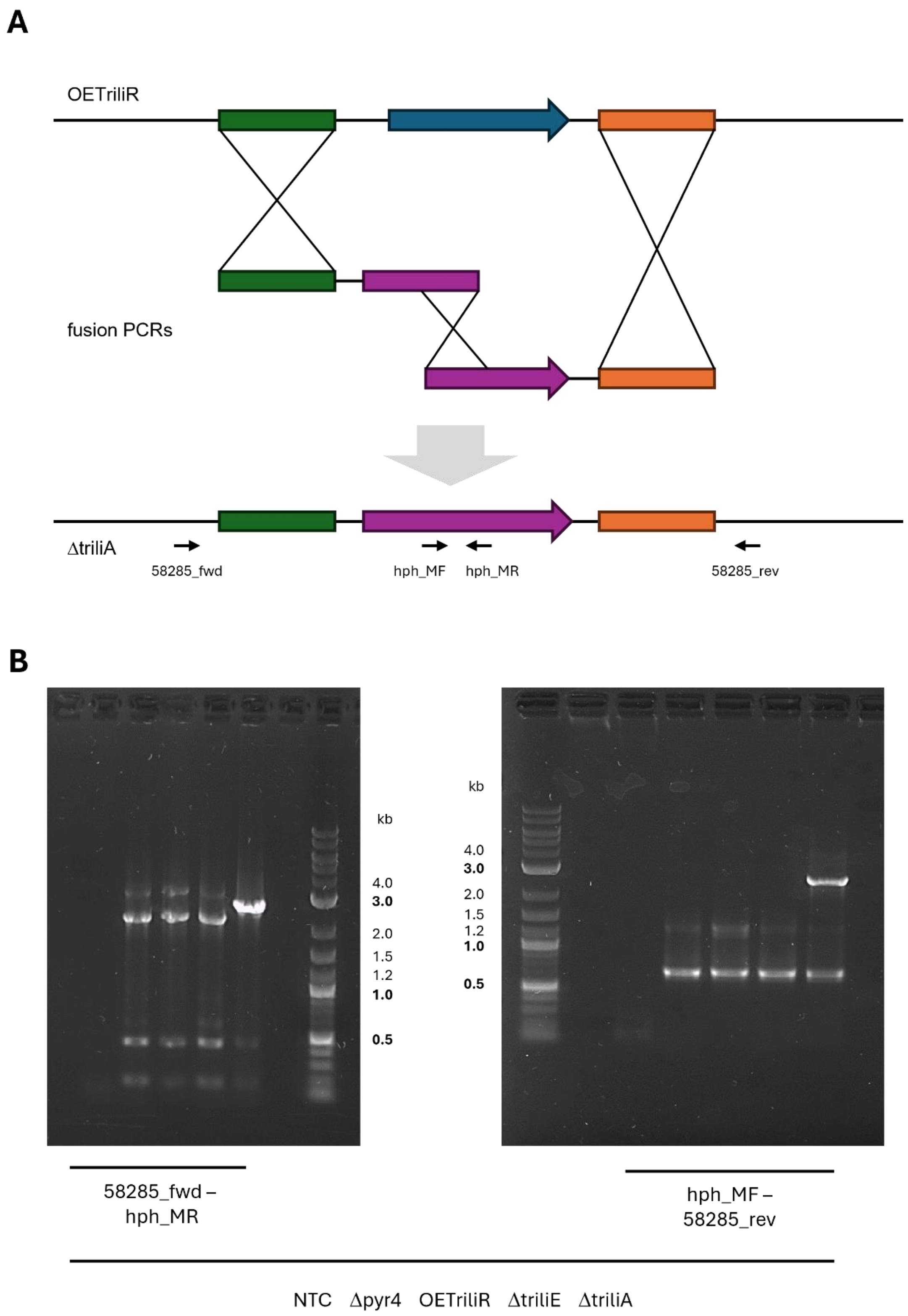

Figure S2. Construction of the strain ΔTriliA. (A) To delete the gene *triliA*, a split marker strategy and the hygromycin resistance marker *hph* were used. (B) The deletion was tested on a genomic level by suitable PCR assays using the indicated primers and the chromosomal DNA of the used strains. NTC, no template control. 1 kb Plus DNA Ladder (NEB) was used for size comparison.

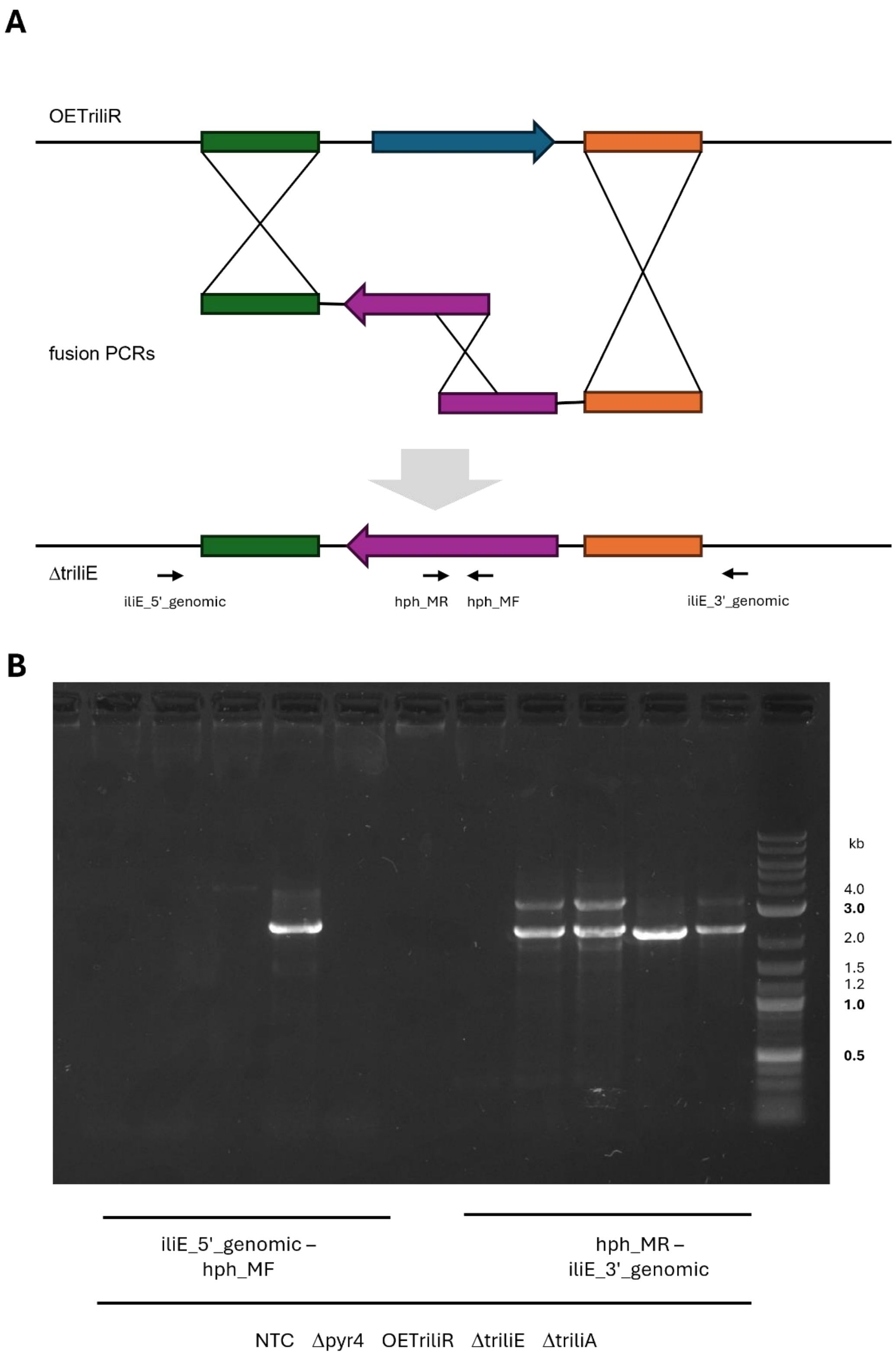

Figure S3. Construction of the strain ΔTriliE. (A) To delete the gene *triliE*, a split marker strategy and the hygromycin resistance marker *hph* were used. (B) The deletion was tested on a genomic level by suitable PCR assays using the indicated primers and the chromosomal DNA of the used strains. NTC, no template control. 1 kb Plus DNA Ladder (NEB) was used for size comparison.

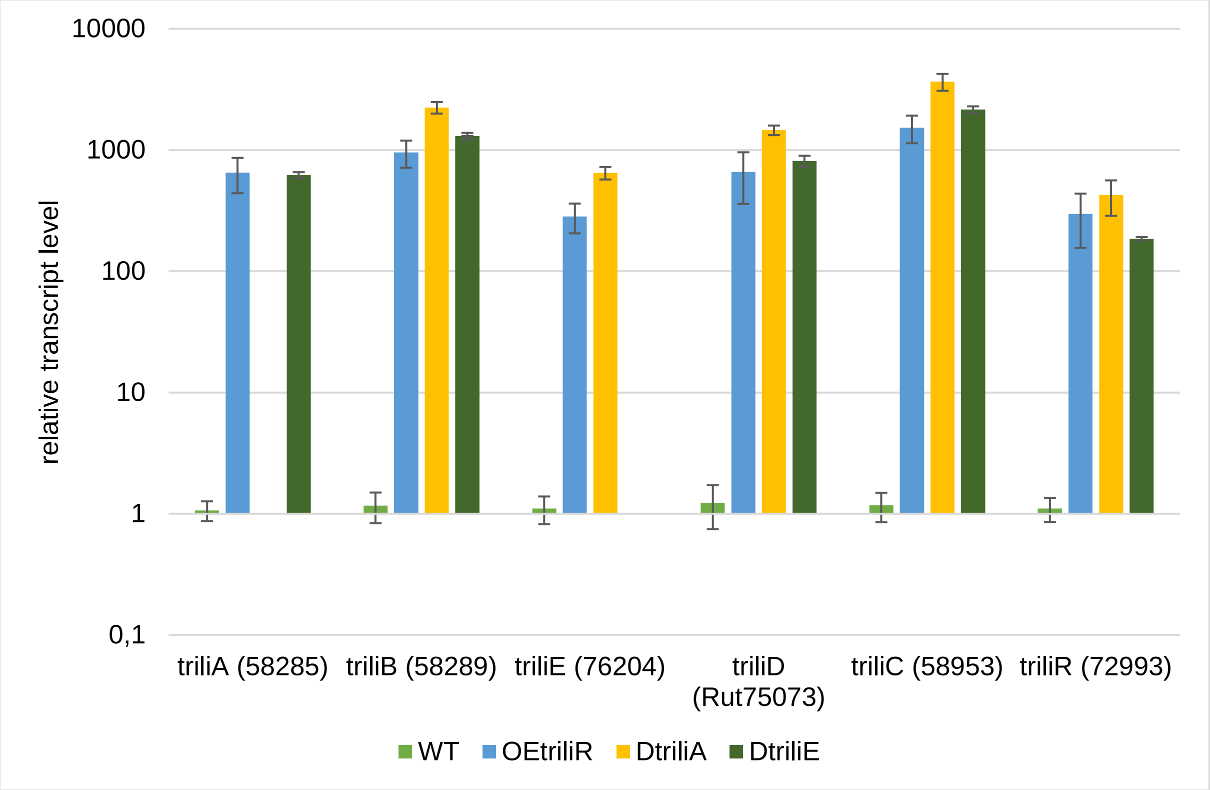

Figure S4. Transcript level analysis using an RT-qPCR assay. The *T. reesei* strains QM6a Δmus53 (wildtype, WT), OETriliR, ΔTriliA, and ΔTriliE were cultivated in MAM with glycerol as carbon source in biological quadruplicates. After 72 hours, the total RNA was extracted, and the cDNA was synthesized using the NEB LunaScript kit. The cDNAs were used as template for the RT-qPCR reactions using the NEB LunaMix according to the manufacturer's instructions. The relative transcript levels of the indicated genes were normalized to the average value of the wildtype samples (WT, QM6a Δmus53) using the Pfaffl[5] method and the genes *sar1* and *act1* as reference genes. Error bars indicate standard deviation.

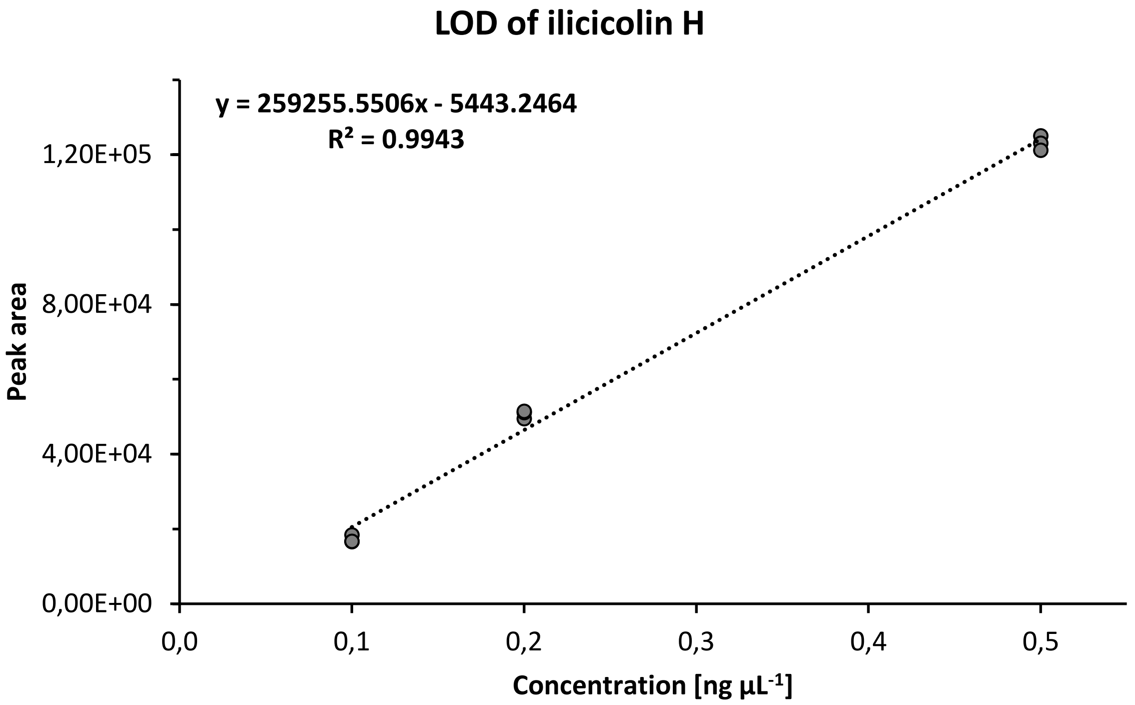

Figure S5. Determination of the limit of detection (LOD) of ilicicolin H using a matrix matched external calibration curve. The timsTOF Pro Mass Spectrometer was set to detect signals with an intensity threshold of I = 100, classifying signals below this threshold as noise. In the extracted ion current chromatograms (EICCs) of ilicicolin H, no signals (defined as having an intensity < 100) were detected in the wildtype and ΔTriliA mycelial extracts. Given that the mycelia contain the compound at higher concentrations, it is reasonable to assume that the media of the wildtype and ΔTriliA strains are practically free of ilicicolin H, due to the dilution effect of the large volume of the medium. Consequently, the media from the wildtype and ΔTriliA were pooled, and low amounts of ilicicolin H standard were spiked into it, to create matrix-matched standard solutions with a concentration of 0.1, 0.2 and 0.5 ng μL^{-1}. The standards were measured in technical triplicates. The resulting peak areas were plotted against the known concentrations to generate a linear regression curve. The derived equation (y = 259255.5506x - 5443.2464) was set equal to the intensity threshold of I = 100, yielding a limit of detection of 0.02138 ng μL^{-1} (= 21.38 ng mL^{-1}).

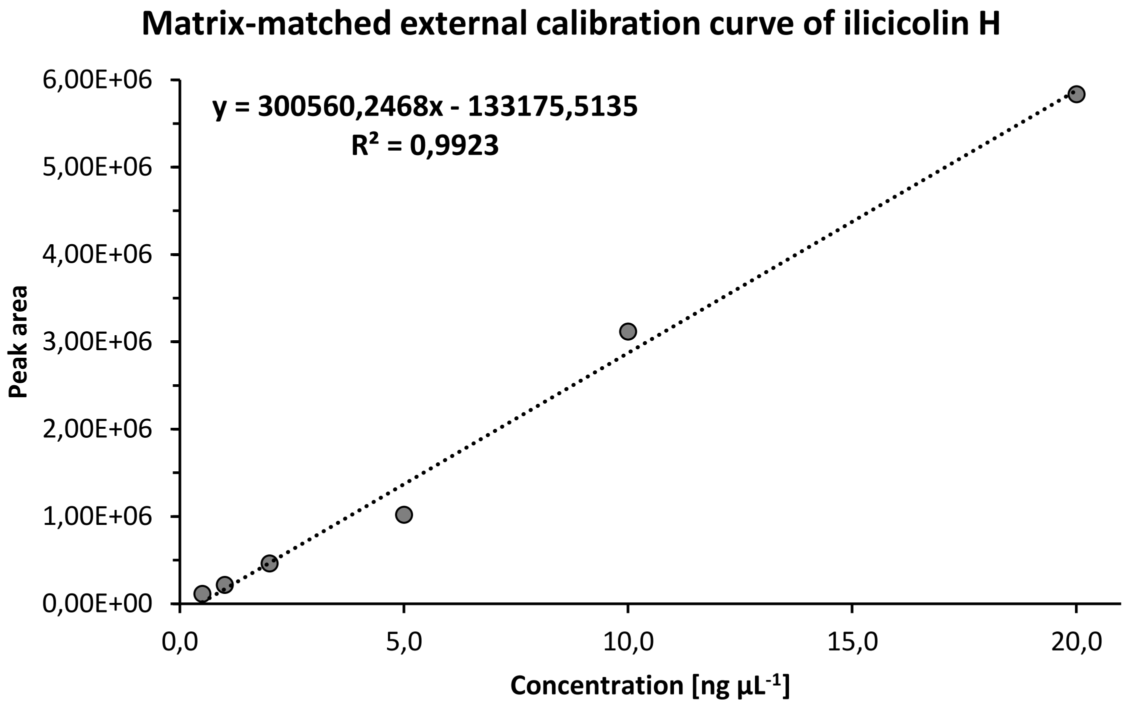

Figure S6. Matrix matched external calibration curve to quantify ilicicolin H in the medium. As matrix, wildtype and ΔTriliA media were pooled. Using the obtained equation (y = 300560.2468x - 133175.5135), the concentration of ilicicolin H was calculated in the samples. (see **Table S2** and **Table S3**).

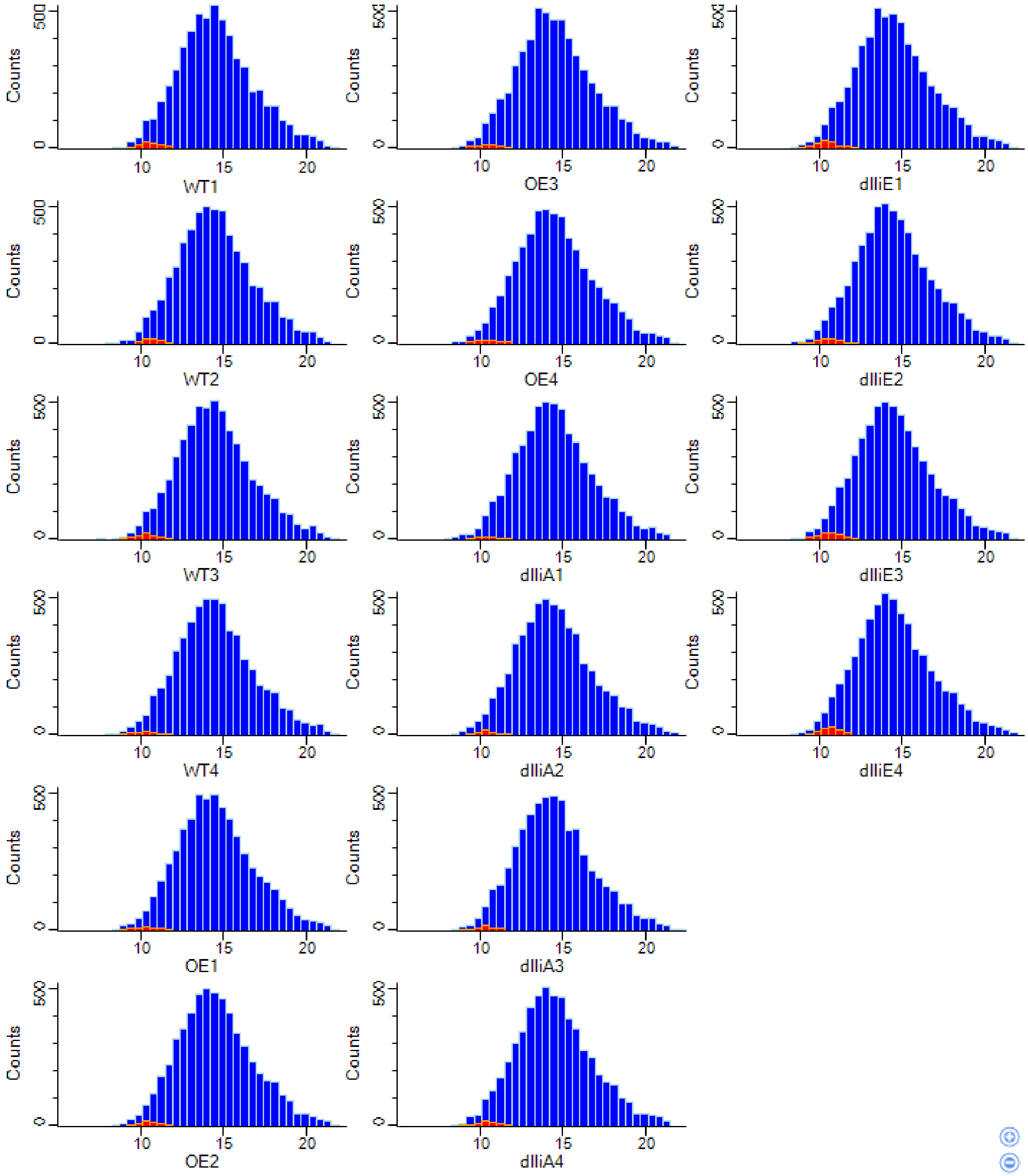

Figure S7. Histograms of proteomics data distribution per each sample. Imputed values are highlighted in red. Proteins were filtered for 4 valid values in at least one group. Imputation of missing values was conducted based on normal distribution (width 0.3, down shift 1.8).

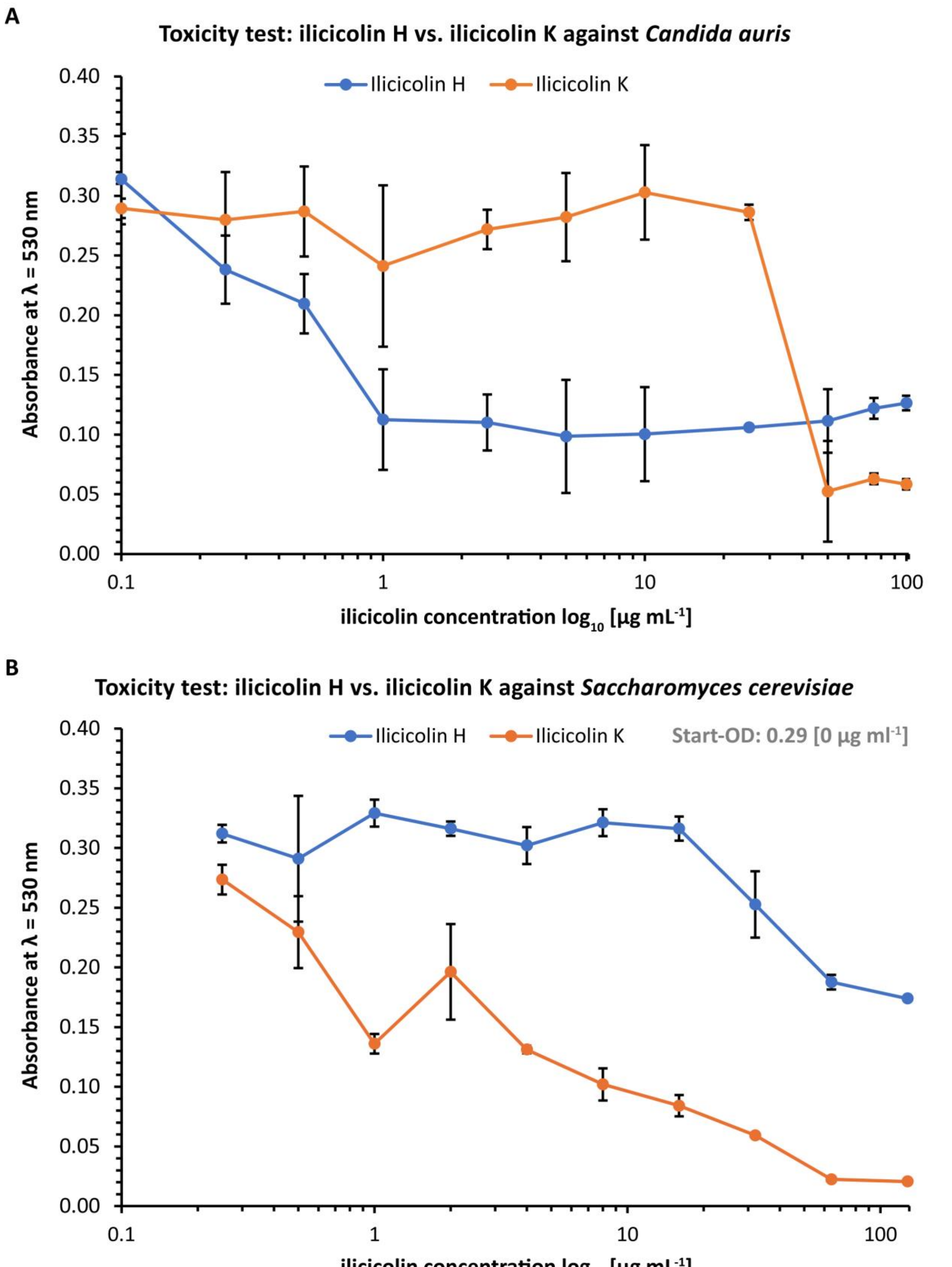

Figure S8. (A) Antifungal activities of ilicicolin H **(1)** and the novel ilicicolin K towards *Candida auris* DSM 21092. (B) Antifungal activities of ilicicolin H **(1)** and the novel ilicicolin K towards *Saccharomyces cerevisiae* CEN.PK113-5D. X-axes were logarithmized for better comparability of the two compounds.

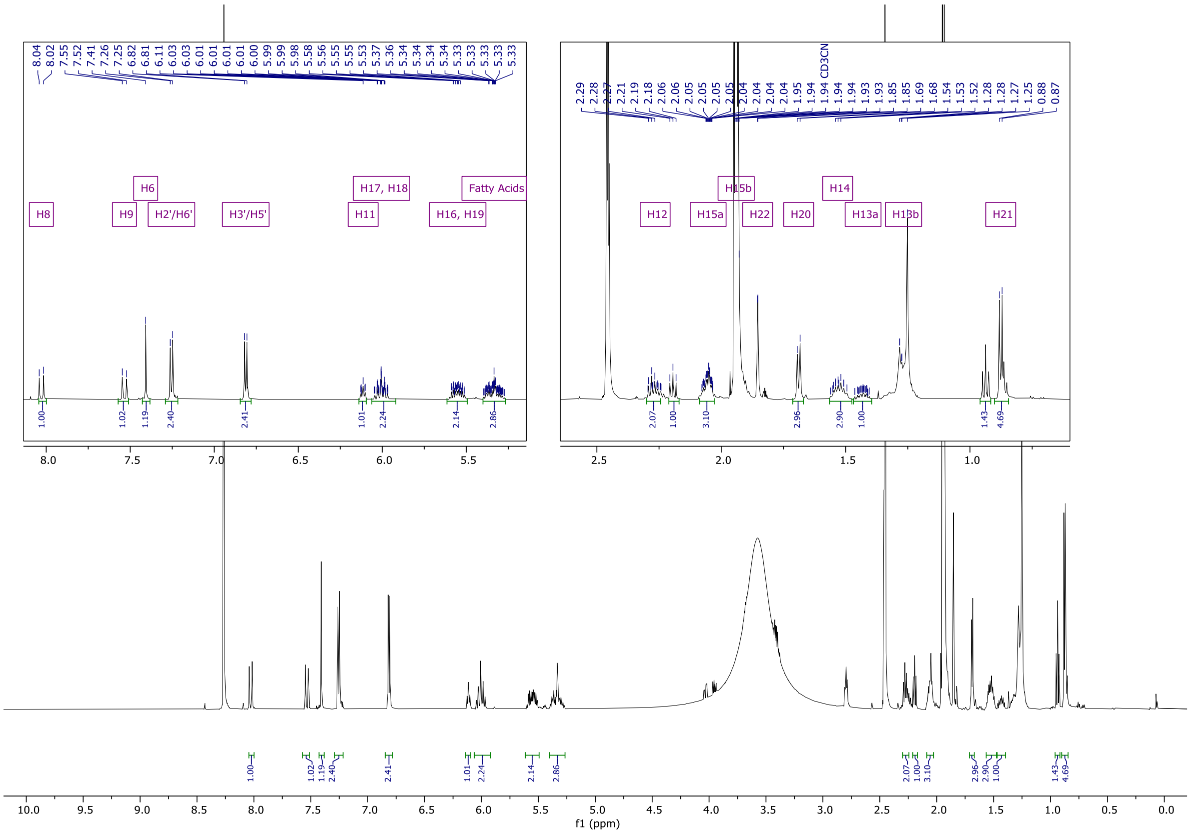

Figure S9. ^{1}H spectrum (600 MHz) of *bis*-diene **(3)** in acetonitrile-*d*3:DMSO (9:1).

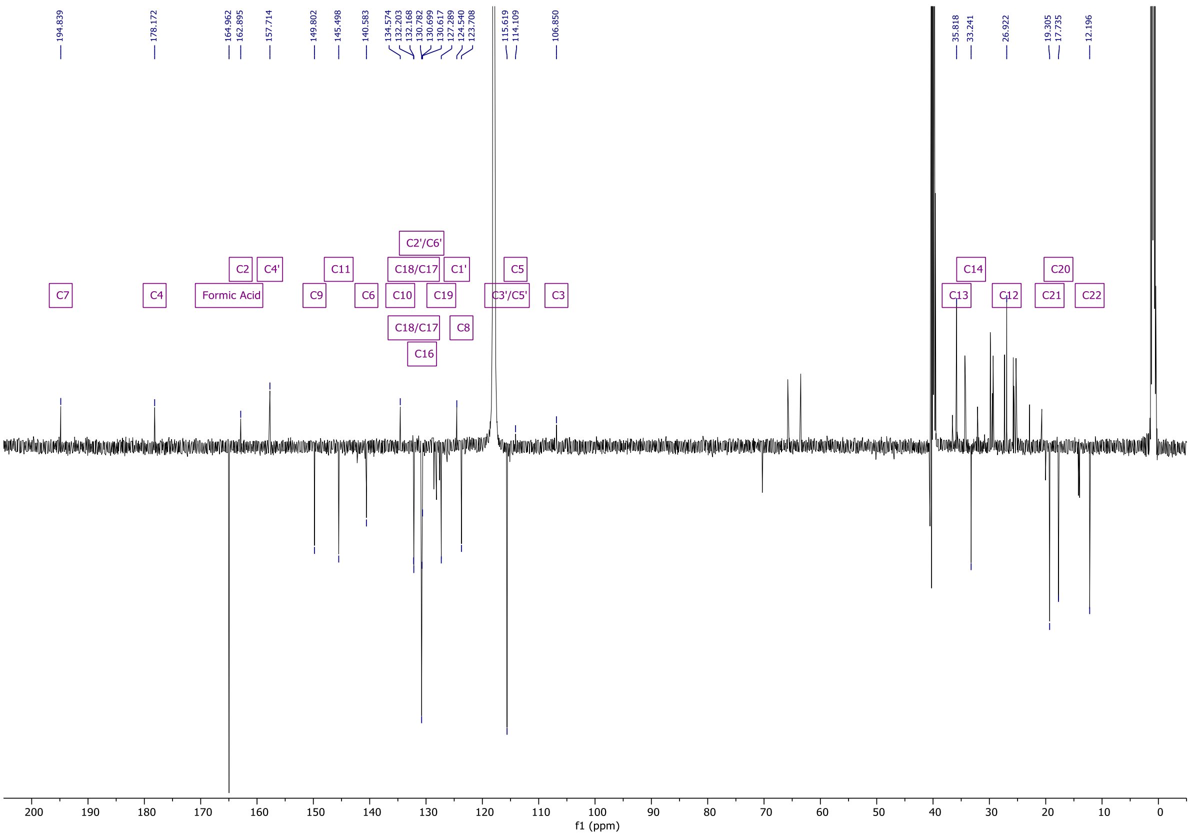

Figure S10. ^{13}C spectrum (151 MHz) of *bis*-diene **(3)** in acetonitrile-*d*3:DMSO (9:1).

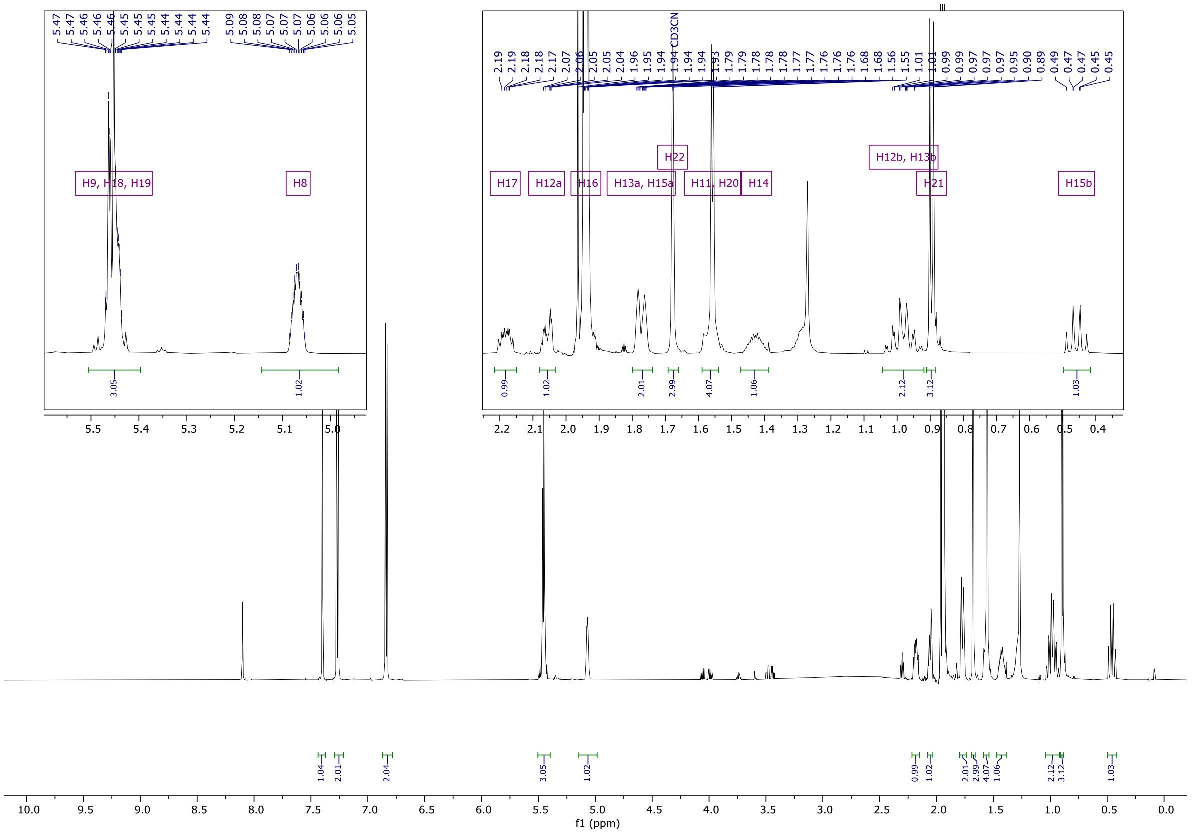

Figure S11. ^{1}H spectrum (600 MHz) of 8-*epi*-ilicicolin H **(2)** in acetonitrile-*d*3.

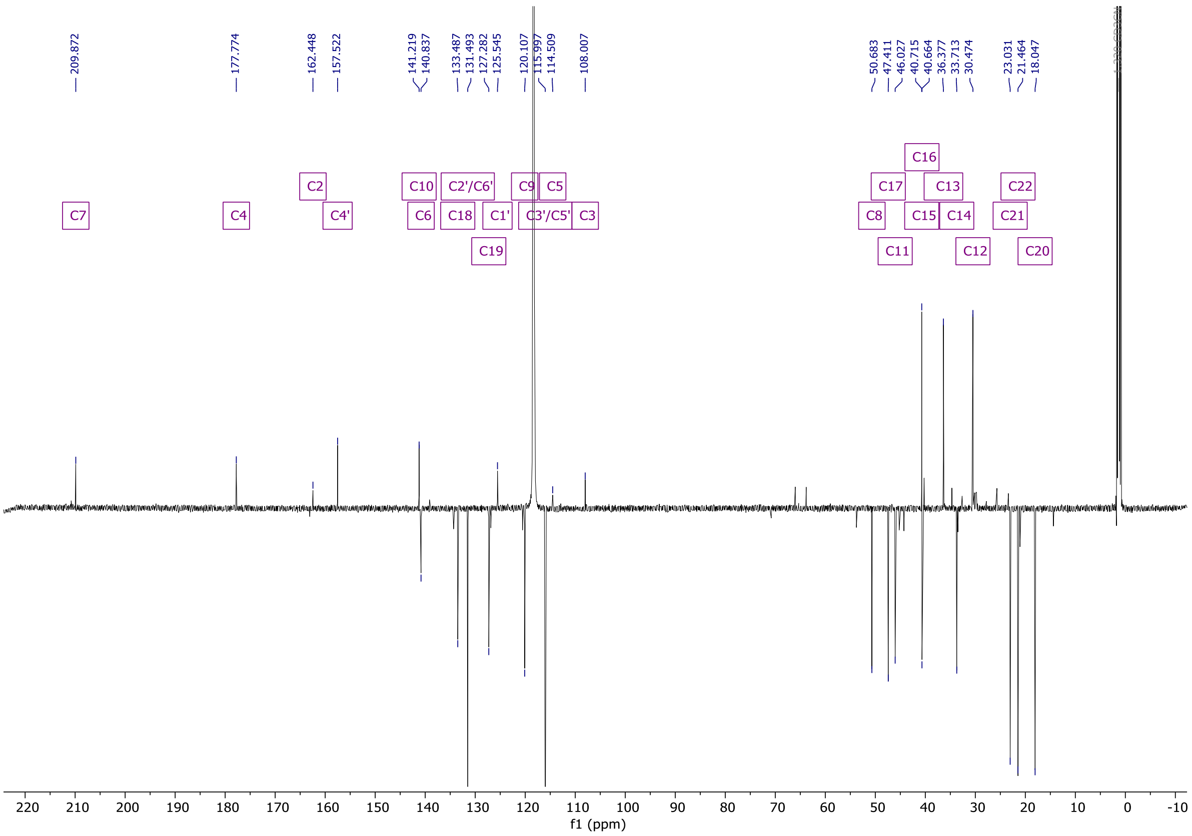

Figure S12. ^{13}C spectrum (151 MHz) of 8-*epi*-ilicicolin H **(2)** in acetonitrile-*d*3.

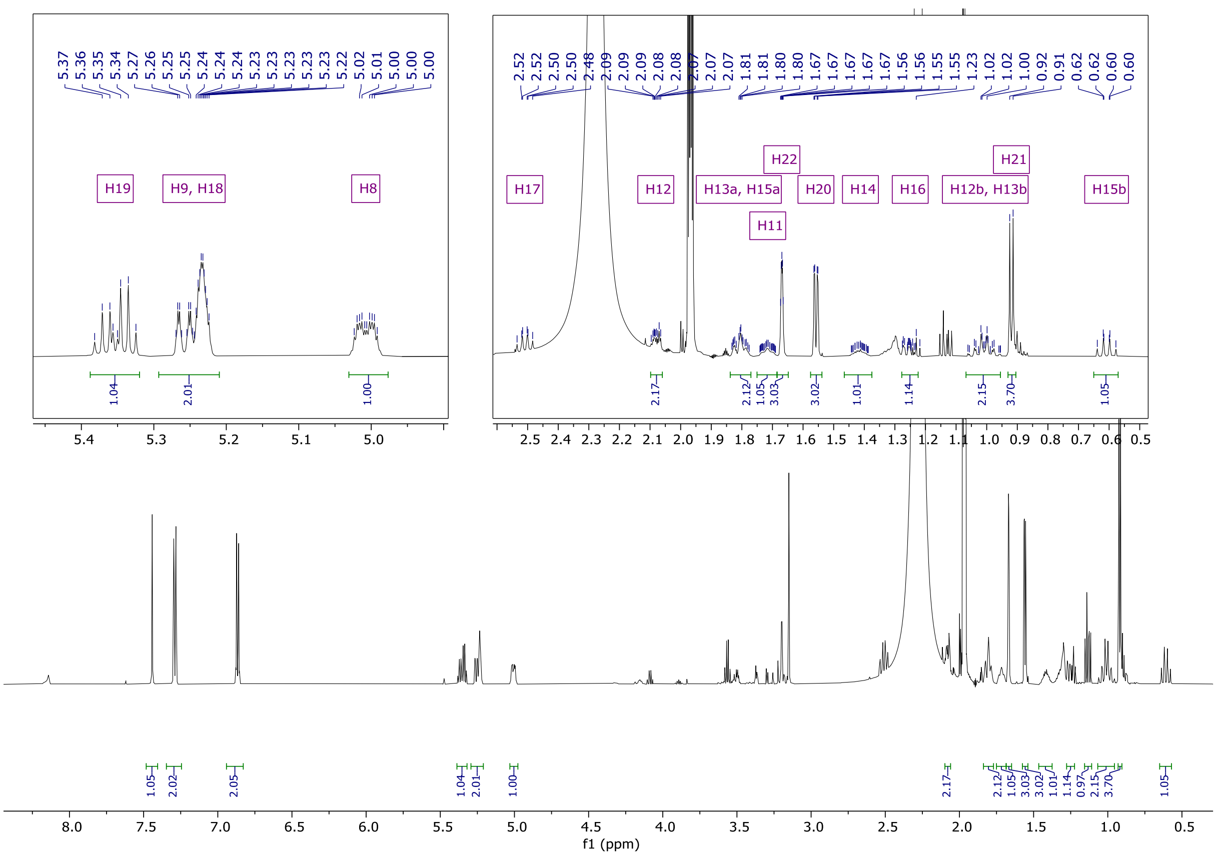

Figure S13. ^{1}H spectrum (600 MHz) of ilicicolin H **(1)** in acetonitrile-*d*3.

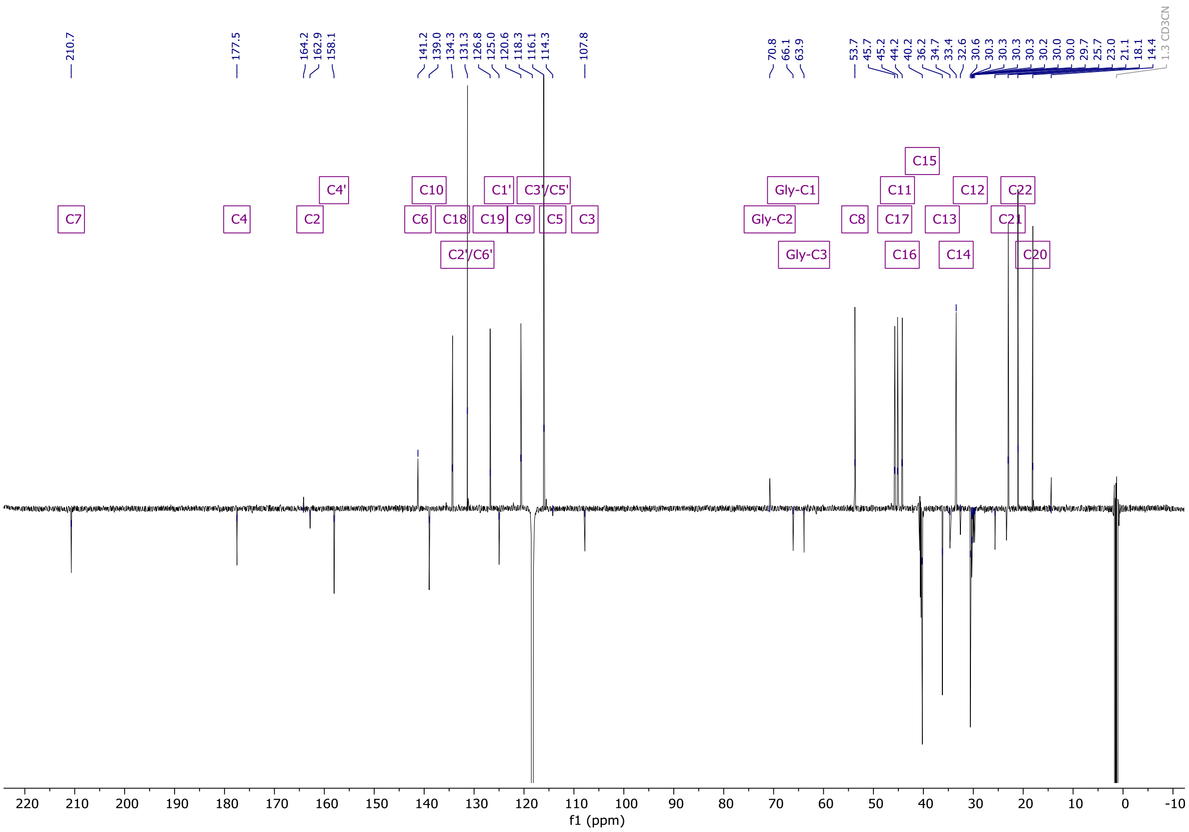

Figure S14. ^{13}C spectrum (151 MHz) of ilicicolin H **(1)** in acetonitrile-*d*3.

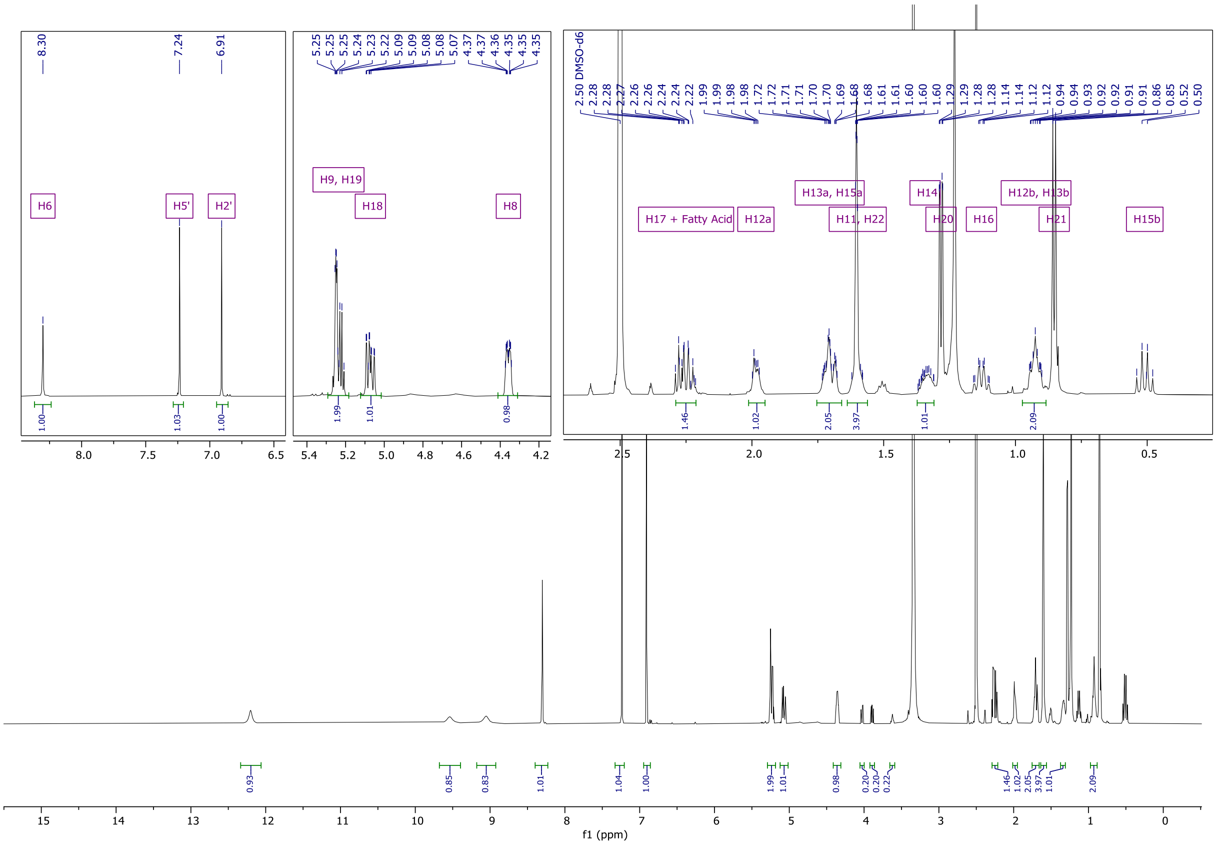

Figure S15. ^{1}H spectrum (600 MHz) of ilicicolin K in DMSO-*d*6.

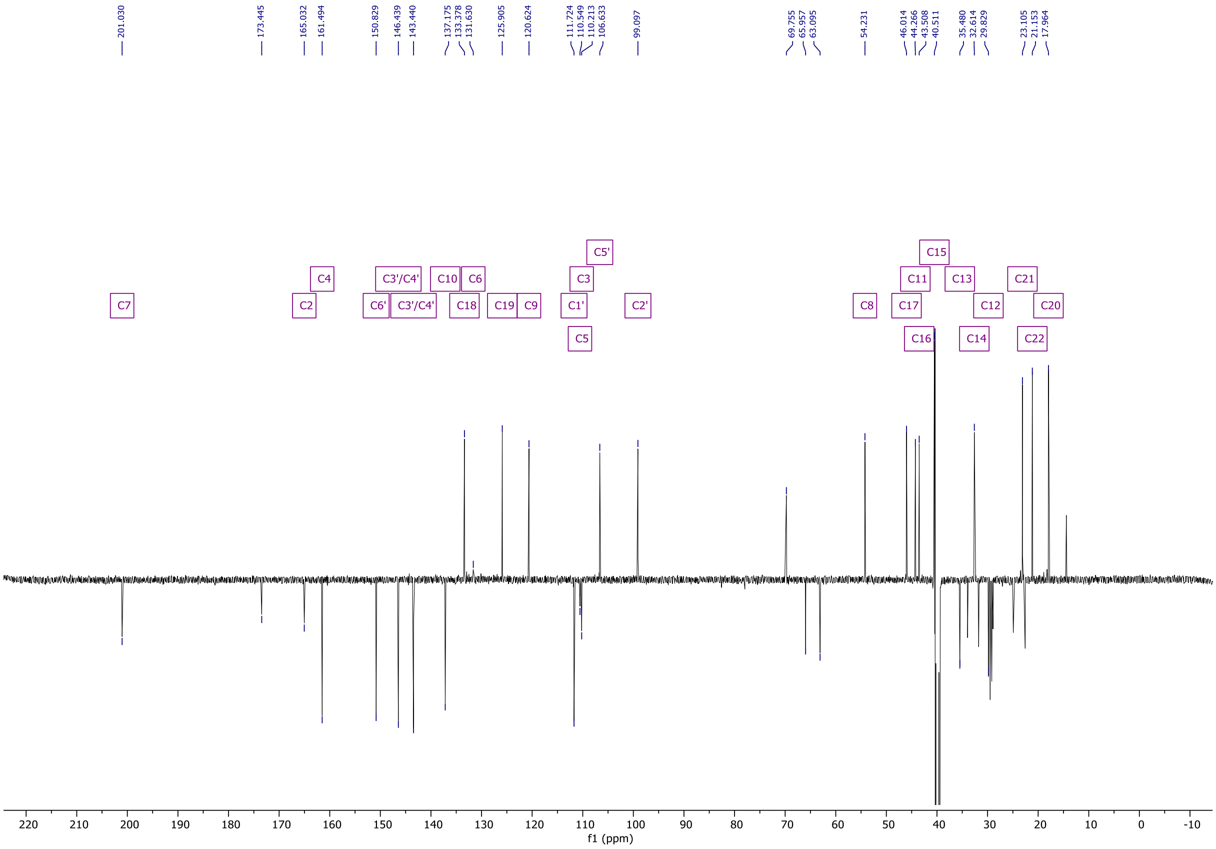

Figure S16. ^{13}C spectrum (151 MHz) of ilicicolin K in DMSO-*d*6.

References

(1) Sikorski, R. S.; Hieter, P. A System of Shuttle Vectors and Yeast Host Strains Designed for Efficient Manipulation of DNA in Saccharomyces Cerevisiae. *Genetics* **1989**, *122* (1), 19–27. https://doi.org/10.1093/genetics/122.1.19.

(2) Zhang, Z.; Jamieson, C. S.; Zhao, Y. L.; Li, D.; Ohashi, M.; Houk, K. N.; Tang, Y. Enzyme-Catalyzed Inverse-Electron Demand Diels-Alder Reaction in the Biosynthesis of Antifungal Ilicicolin H. *J Am Chem Soc* **2019**, *141* (14), 5659–5663. https://doi.org/10.1021/jacs.9b02204.

(3) Todorov, A. R.; Wirtanen, T.; Helaja, J. Photoreductive Removal of O -Benzyl Groups from Oxyarene N -Heterocycles Assisted by O -Pyridine–Pyridone Tautomerism. *J Org Chem* **2017**, *82* (24), 13756–13767. https://doi.org/10.1021/acs.joc.7b02775.

(4) Derntl, C.; Kiesenhofer, D. P.; Mach, R. L.; Mach-Aigner, A. R. Novel Strategies for Genomic Manipulation of Trichoderma Reesei with the Purpose of Strain Engineering. *Appl Environ Microbiol* **2015**, *81* (18), 6314–6323. https://doi.org/10.1128/AEM.01545-15.

(5) Pfaffl, M. W. A New Mathematical Model for Relative Quantification in Real-Time RT–PCR. *Nucleic Acids Res* **2001**, *29* (9), e45–e45. https://doi.org/10.1093/NAR/29.9.E45.
